# Network States Classification based on Local Field Potential Recordings in the Awake Mouse Neocortex

**DOI:** 10.1523/ENEURO.0073-22.2022

**Published:** 2022-08-19

**Authors:** Yann Zerlaut, Stefano Zucca, Tommaso Fellin, Stefano Panzeri

**Affiliations:** 1 Neural coding laboratory, Istituto Italiano di Tecnologia, 16163 Genova, Italy; 2 Neural Computation Laboratory, Center for Neuroscience and Cognitive Systems @UniTn, Istituto Italiano di Tecnologia, 38068 Rovereto, Italy; 3 Optical Approaches to Brain Function Laboratory, Istituto Italiano di Tecnologia, 16163 Genova, Italy; 4 Paris Brain Institute (Institut du Cerveau et de la Moelle épinière; ICM), Institut National de la Santé et de la Recherche Médicale, Centre National de la Recherche Scientifique, Assistance Publique Hôpitaux de Paris, Hôpital de la Pitié Salpêtrière, Sorbonne Université, 75013 Paris, France; 5 Department of Excellence for Neural Information Processing, Center for Molecular Neurobiology (ZMNH), University Medical Center Hamburg-Eppendorf, 20251 Hamburg, Germany

**Keywords:** cortex, intracellular recordings, local field potential, state-dependent processing, waking states

## Abstract

Recent studies using intracellular recordings in awake behaving mice revealed that cortical network states, defined based on membrane potential features, modulate sensory responses and perceptual outcomes. Single-cell intracellular recordings are difficult and have low yield compared to extracellular recordings of population signals, such as local field potentials (LFPs). However, it is currently unclear how to identify these behaviorally-relevant network states from the LFP. We used simultaneous LFP and intracellular recordings in the somatosensory cortex of awake mice to design a network state classification from the LFP, the Network State Index (NSI). We used the NSI to analyze the relationship between single-cell (intracellular) and population (LFP) signals over different network states of wakefulness. We found that graded levels of population signal faithfully predicted the levels of single-cell depolarization in nonrhythmic regimes whereas, in δ ([2–4 Hz]) oscillatory regimes, the graded levels of rhythmicity in the LFP mapped into a stereotypical oscillatory pattern of membrane potential. Finally, we showed that the variability of network states, beyond the occurrence of slow oscillatory activity, critically shaped the average correlations between single-cell and population signals. Application of the LFP-based NSI to mouse visual cortex data showed that this index increased with pupil size and during locomotion and had a U-shaped dependence on population firing rates. NSI-based characterization provides a ready-to-use tool to understand from LFP recordings how the modulation of local network dynamics shapes the flexibility of sensory processing during behavior.

## Significance statement

Sensation during behavior is strongly modulated by the animal’s internal state. Such context-dependent modulation of sensory processing is believed to largely stem from top-down control of network states in sensory cortices, with different network states being associated with distinct computational properties of the circuit. So far, a detailed characterization of network states in the awake cortex has mostly been achieved through single-cell intracellular recordings, which however cannot be easily recorded. Here, we developed a new method to classify network states from the easily accessible extracellular LFP recordings of population activity. Given the widespread use of LFPs, our work provides a critical methodology to greatly expand our understanding of the mechanisms underlying state-dependent computations in neocortex.

## Introduction

While there is a long history of using extracellular and scalp signals to characterize brain states (Berger, 1929; Creutzfeldt et al., 1966; Steriade et al., 1990, 1993), recent studies have shown that the membrane potential of individual neurons, which can be measured via intracellular recording techniques, can serve as a particularly sensitive indicator for neural network activity. Intracellular recordings have been instrumental to characterize network states in unprecedented details, defining them as a set of distinctive dynamical features that include oscillatory patterns and depolarization levels. In particular, in awake rodents these studies have correlated variations of behavioral and physiological markers, such as pupil diameter, whisking activity, and locomotion speed with distinct membrane potential dynamics in sensory cortices (Crochet and Petersen, 2006; Poulet and Petersen, 2008; Okun et al., 2010; Constantinople and Bruno, 2011; Bennett et al., 2013; Polack et al., 2013; Reimer et al., 2014; Schneider et al., 2014; McGinley et al., 2015a; Schiemann et al., 2015; Einstein et al., 2017; Neske et al., 2019; Poulet and Crochet, 2019; Nestvogel and McCormick, 2022). A powerful concept for the modulation of network activity in sensory cortices emerging form these studies is a “U-model” of network states (McGinley et al., 2015b). This model is based on the observation that, in some sensory detection tasks, performance depends on the arousal level following an inverted U-shape (it is maximal at intermediate arousal levels (McGinley et al., 2015a; Neske et al., 2019). This model posits the existence of a continuum of dynamical network states across arousal levels which includes three major and well-documented patterns of membrane potential fluctuations. At low arousal levels (small pupil diameter and absence of motor behavior), membrane potential fluctuations largely exhibit stereotypical δ-band oscillations. At moderate arousal (intermediate pupil diameter), single cells are hyperpolarized and display low-amplitude membrane potential fluctuations. At high arousal levels (active motor behavior and/or large pupil dilation), the membrane potential exhibits sustained depolarization with high-frequency fluctuations and high firing activity occurs. Network states identified based on these properties of membrane potential dynamics profoundly modulate perceptual abilities and cortical processing of sensory stimuli (McGinley et al., 2015a; Neske et al., 2019).

These results shed light on how the internal state of the animal modulates sensory information processing about external stimuli. However, in many experimental settings using awake animals, extracellular measurement of population-level LFP signals is often preferred over single-cell intracellular recordings, because of low yield and high technical difficulty of intracellular experiments. Although LFPs capture subthreshold and integrative phenomena in a local neuronal population (Buzsáki et al., 2012; Panzeri et al., 2015), it is currently unknown how to identify the variety of network states previously described with membrane potentials from the LFP. Furthermore, while it has been reported that single-cell membrane potentials and population-level LFPs are related and their relation varies considerably across cortical states and behavioral conditions (Poulet and Petersen, 2008; Okun et al., 2010; Neske et al., 2019; Nestvogel and McCormick, 2022), it is not yet fully clear how to predict when they are tightly related and when they are not. Precise classification of network state variability from LFPs and its comparison with network state classification performed on membrane potentials could thus be greatly useful to enhance our understanding of how network states change during behavior and what function they may serve. Moreover, such a classification would enhance our comprehension of the relationship between single-cell and population dynamics.

By combining simultaneous intracellular and extracellular recordings in the somatosensory cortex of awake head-fixed mice with novel analytical methods, here we developed an approach to identify, from the LFP signal alone, low-frequency rhythmic states as well as nonrhythmic network states with different levels of depolarization or hyperpolarization. We first characterized the membrane potential dynamics across different cortical states in awake mice. We then identified the LFP properties that better distinguished network states and we used those LFP properties to derive a method for robust classification of network states. Next, we show that our classification method enables to classify well network states and explains the variability of the relationship between LFPs and membrane potentials observed across recordings of neural activity during wakefulness. Finally, we illustrate the generality of the method by applying the NSI classification on recordings from the “Visual coding – Neuropixels” dataset shared by the Allen Institute (Siegle et al., 2021).

## Materials and Methods

### Animals

Experimental procedures involving animals have been approved by the IIT Animal Welfare Body and by the Italian Ministry of Health (authorization # 34/2015-PR and 125/2012-B), in accordance with the National legislation (D.Lgs. 26/2014) and the European legislation (European Directive 2010/63/EU). Experiments were performed on young-adult (four to six weeks old, either sex) C57BL/6J mice (Charles River). The animals were housed in a 12/12 h light/dark cycle in singularly ventilated cages, with access to food and water *ad libitum*.

### Experimental design

The experimental procedure for simultaneous extracellular and intracellular recordings in awake head-fixed mice have been previously described (Zucca et al., 2017), and the present dataset was used in a previous study (Zerlaut et al., 2019). Briefly, a custom metal plate was fixed on the skull of *n* = 4 young (22-24 post-natal days) mice two weeks before the experimental sessions. After a 2- to 3-d recovery period, mice were habituated to sit quietly on a fixed platform (without the ability to freely run) for at least 7–10 d (one session per day and gradually increasing session duration). Mice were not involved in any task, and whisking and pupil size were not measured. The day of the experiment, mice were anesthetized with 2.5% isoflurane and a small craniotomy (0.5 x 0.5 mm) was opened over the somatosensory cortex. A 30-min-long recovery period was provided to the animal before starting recordings. Brain surface was kept moist with a HEPES-buffered artificial CSF (aCSF). Local field potential (LFP) recordings were performed by lowering a glass pipette filled with aCSF into the tissue with the tip placed at ∼300 μm from pial surface. Simultaneous current-clamp patch-clamp recordings were carried out on superficial layers (100–350 μm) within the same craniotomy. The distance between the tips of the electrodes was in the 200- to 250-μm range. All recorded cells had a regular-spiking response to current pulses (data not shown) and were therefore identified as putative pyramidal neurons (Connors and Gutnick, 1990); 3–6 M Ω borosilicate glass pipettes (Hilgenberg) were filled with an internal solution containing (in mm): 140 K-gluconate, 1 MgCl_2_, 8 NaCl, 2 Na2ATP, 0.5 Na3GTP, 10 HEPES, and 10 Tris-phosphocreatine to pH 7.2 with KOH. Current-clamp recordings were not corrected for liquid junction potential offset. Electrical signals were grounded at the top of the skull (at the location of the craniotomy) and were acquired using a Multiclamp 700B amplifier, filtered at 10 kHz, digitized at 50 kHz with a Digidata 1440 and stored with pClamp 10 (Axon Instruments). From the previously described recordings, we extracted samples with stable membrane potential periods. The early hyperpolarized period (∼5 min) following pipette lowering was discarded from the analysis. Next, periods with action potential peaking below 0 mV or displaying a slow (∼1 min) drift in the *V_m_* trace were discarded from the analysis. This criterion enabled us to perform the analysis on an absolute scale of membrane potential values. Multiunit activity (MUA) was computed by band-pass filtering (0.3–3 kHz) the extracellular signal and taking the absolute value of the resulting signal (Einevoll et al., 2013).

### Wavelet transform

Our signal processing pipeline of the LFP was based on the wavelet transform. We implemented a wavelet transform based on the Morlet wavelet, which has the following equation (Torrence and Compo, 1998):

(1) $$M_{d_{0}}\left(f,t\right)=C_{d_{0}}\left(f\right)\cdot e^{2i\pi ft}\cdot e^{-{\left(\frac{\sqrt{2}\pi ft}{d_{0}}\right)}^{2}},$$

where f is the frequency of the wavelet and $d_{0}$ the decay parameter of the envelope. We used a value of $d_{0}$=6 throughout the study. The coefficient $C_{d_{0}}\left(f\right)$ is the normalization coefficient of the wavelet. Note that, to keep a meaningful link with the physical units of the signal, we did not normalize the wavelet with respect to itself, but with respect to a sinusoid (otherwise the wavelet transform with standard normalization of a sinusoid of frequency f and amplitude 1 has a value greater than 1 at the frequency f). The wavelet normalization coefficient was therefore defined, for a wavelet frequency f and an extent *d*_0_, as:

$$C_{d_{0}}\left(f\right)=\overset{\infty }{\underset{-\infty }{\int }}cos\left(2\pi fs\right)\cdot \bar{M_{d_{0}}\left(f,s\right)}ds=\frac{d_{0}}{2\sqrt{2\pi }f}\left(1+e^{\frac{-d_{0}^{2}}{2}}\right).$$

The transform was implemented by a convolution between the complex conjugate of the Morlet wavelet (Eq. 1) and the signal S(t), i.e.:

(2) $$W\left(f,t\right)=\overset{T_{f}}{\underset{-T_{f}}{\int }}\left(S\left(t-s\right)-{〈S〉}_{t,T_{f}}\right)\cdot \bar{M_{d_{0}}\left(f,s\right)}\;ds,$$

where ${〈S〉}_{t,T_{f}}$ is the signal average in the window centered at t of extent $T_{f}$. $T_{f}$ is the frequency-dependent window on which the convolution is performed, it was defined as the extent of the wavelet where its amplitude decays by $\left(1-e^{-4}\right)$ = 98.2%, i.e., $T_{f}=\sqrt{2}\frac{d_{0}}{\pi f}$. From Equation 2, we computed the envelope and the phase at time t of a given frequency f in the signal by taking the norm and argument of the complex number W(f,t).

### Computing the pLFP signal

**Figure 1.**
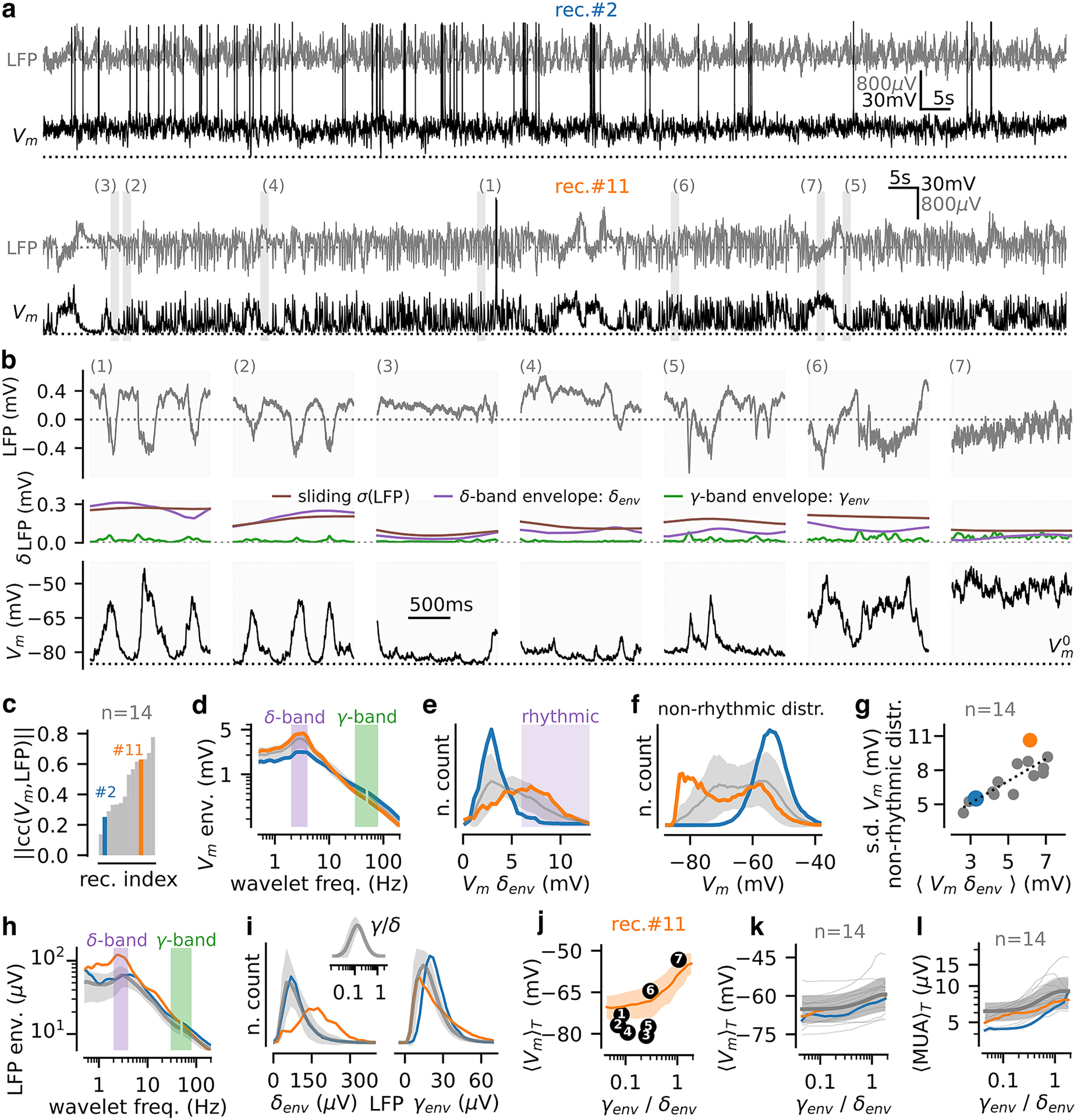
Network states of wakefulness in the mouse somatosensory cortex: electrophysiological signature and characterization based on spectral analysis. ***a***, Two examples of simultaneous recordings (top: recording #2, bottom: recording #11) of the extracellular LFP and of the membrane potential V_m_ of a Layer II/III pyramidal cell in awake mouse. ***b***, Episodes of duration 1.5 s extracted from recording #11 at the times points highlighted in **a** (LFP on top and V_m_ on the bottom panel). In the middle panel, the time-varying SD σ(LFP) evaluated over a 500-ms sliding window (brown line), the δ-envelope δ_env_ (purple line), and the γ envelope γ_env_ (green line) of the fluctuations are shown. ***c***, Sorted histograms of the absolute correlation coefficient between LFP and V_m_ over recordings (*n* = 14, gray). Blue (recording #2) and orange (recording #11) indicate the example recordings shown in ***a***. ***d***, Frequency spectrum of the V_m_ signal obtained with wavelet-based time-frequency analysis (see Materials and Methods). The power-line frequency was blanked (50 ± 2 Hz). We highlighted the δ (2–4 Hz) and γ (30–80 Hz) bands in purple and green, respectively. ***e***, Histogram of V_m_ δ envelope across recordings. Time samples classified as “rhythmic” are shown in purple (see main text). ***f***, Histogram of V_m_ depolarization level for nonrhythmic samples. ***g***, Relationship between mean δ envelope of the V_m_ and SD in nonrhythmic episodes per recording for all recordings. ***h***, Same as in ***d*** for the extracellular LFP. ***i***, Histogram of the δ envelope (δ_env_, left) and the γ envelope (γ_env_, right) of the LFP over recordings. In the top inset, we show the histogram of the resulting γ-to-δ ratio. ***j***, Mean depolarization level (shown as mean ± SEM over time samples at a given γ-to-δ level) as a function of the γ-to-δ ratio for a single recording (recording #11, shown in ***a***, ***b***). We highlight how the γ-to-δ measure classifies the episodes shown in ***b*** (see main text). ***k***, Mean depolarization level as a function of the γ-to-δ ratio for recording #11. ***k***, Mean depolarization level as a function of the γ-to-δ ratio over time samples. ***l***, Relationship between MUA (see Materials and Methods) and γ-to-δ ratio over time samples. For panels ***d****–****f***, ***h***, ***i***, ***k***, ***l***, we show two example recordings (recording #2 in blue and recording #11 in orange) and the population data as mean ± SEM. over *x*-axis levels (*n* = 14, gray line with shaded area). See Extended Data Figure 1-1 for the relationship between cellular properties and V_m_-LFP correlations across recordings.

From the LFP time series (Fig. 1), we computed a “processed LFP” (pLFP; Mukovski et al., 2007), which corresponds to the temporally-smoothed high-γ envelope variations of the LFP fluctuations (see main text). The pLFP was computed as follows. We consider a frequency band spanning [ $f_{0}/w_{0}$, $f_{0}\cdot w_{0}$], where $f_{0}$ is a root frequency and $w_{0}$ the width parameter of the band. We take a set of N = 5 wavelets uniformly spanning this band (i.e., evenly space from $f_{0}/w_{0}$ to $f_{0}\cdot w_{0}$). The pLFP signal was computed as the sum over N of the k wavelet envelopes of frequency $f_{k}\in \left[f_{0}/w_{0},f_{0}\cdot w_{0}\right]$, i.e.:

(3) $$pLFP\left(t\right)=\overset{}{\underset{k\in \left[1,N\right]}{\sum }}\frac{∥W\left(f_{k},t\right)∥}{N}.$$

**Figure 2.**
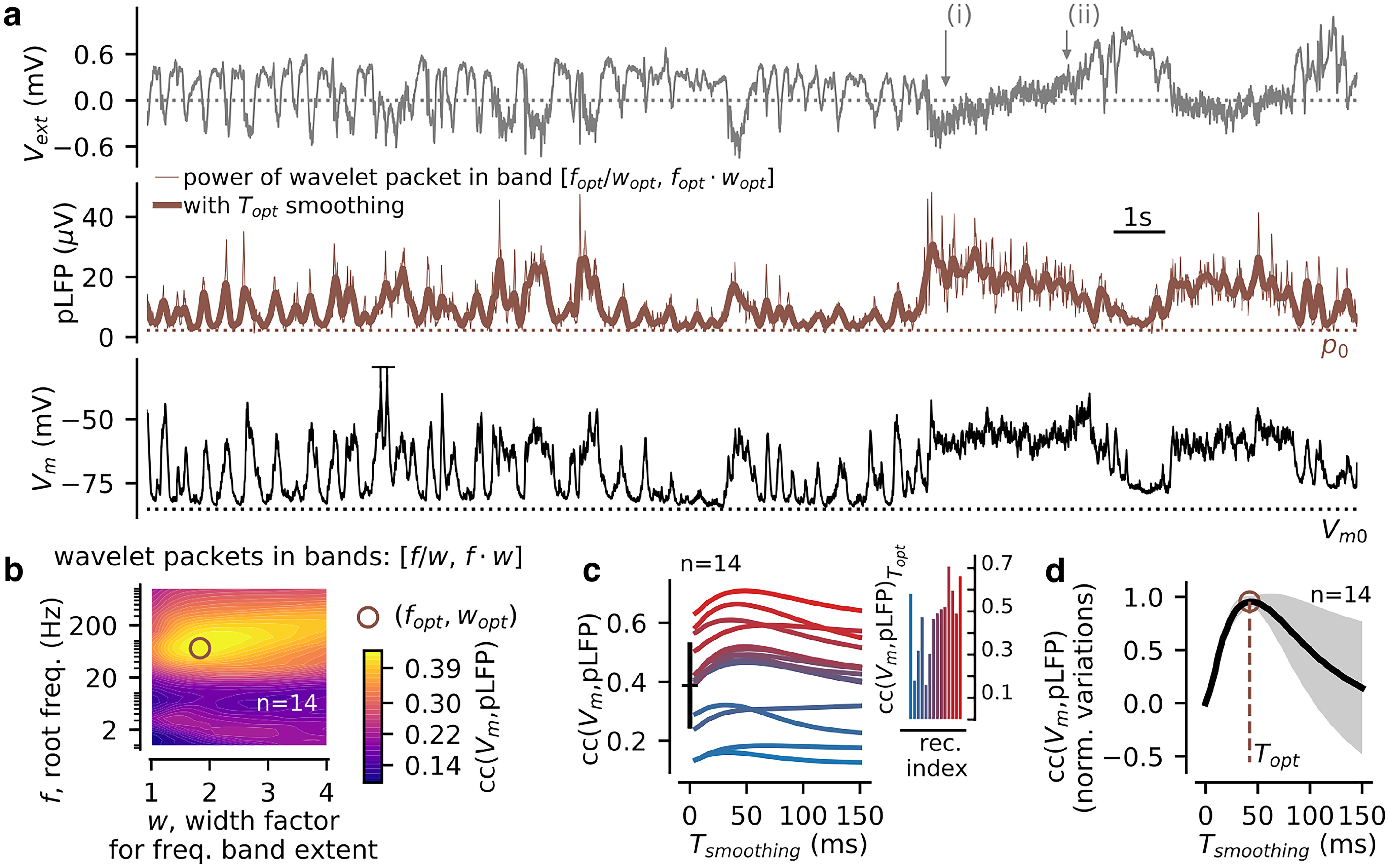
The time-varying high-γ envelope of the LFP displays strong correlations with the membrane potential of pyramidal neurons in awake mice S1. ***a***, Example simultaneous recording of the LFP (top) and V_m_ of a layer 2/3 pyramidal cell (bottom). In the middle, we show the time-varying high-γ envelope (brown thin line) and its smoothed fluctuations (brown thick line, the pLFP signal). The *p_0_* value (brown dotted line) corresponds to the first 100th percentile of the pLFP distribution over the whole recording. ***b***, Cross-correlation between V_m_ and the envelope of the LFP wavelet transform in the frequency band [*f/w, f·w*] (*f* is a root frequency and *w* is a width factor). We show the cross-correlation value after averaging over *n* = 14 recordings (see the individual values per recording in ***c***). Note the optimal band found for *f_opt_* = 72.8 Hz and *w_opt_* = 1.83 (brown circle). ***c***, The effect of temporal smoothing on the cross-correlation between the LFP and *V_m_* signals. Shown for all recordings [individual recordings are color-coded according to their cc(V_m_,pLFP) value, we show the correspondence with the recording index of Fig. 1*c* in the inset]. At *T_smoothing_* = 0 ms, one can see the mean value of ***b*** and its variability over recordings (black error bar). ***d***, Cross-correlation between LFP and *V_m_* as function of the temporal smoothing parameter plotted after normalizing the raw cross-correlation levels of **c** by their maximum amplitude and subtraction of their level at *T_smoothing_* = 0 ms. With this normalization, a peak is clearly visible at *T_opt_* = 42.2 ms.

This time-varying signal is then smoothed over time with a Gaussian filter to yield the final pLFP signal (see Fig. 2*a*). The parameters $f_{0}$, $w_{0}$, and the time smoothing width

10.1523/ENEURO.0073-22.2022.f1-1 Extended Data Figure 1-1 Relationship between cellular properties and V_m_ –LFP correlations across recordings. ***a***, Relationship between LFP-V_m_ correlation and input resistance. The input resistance was estimated after fitting the response to a subthreshold current step stimulation to the response of a RC circuit. ***b***, Relationship between LFP-V_m_ correlation and action potential threshold. The action potential threshold level was estimated at the lowest current level leading to action potential generation in a FI-curve protocol. Download Figure 1-1, TIF file.

$T_{smooth}$ were set to maximize the correlations between the time course of the membrane potential and the pLFP (see Results) and their value is reported in Table 1.

**Table 1:** Parameters of the NSI_pLFP_ characterization

Parameter	Symbol	Value
δ Band	*F*_δ_	[2,4] Hz
pLFP root frequency	$f_{0}$	72.8 Hz
pLFP band factor	$w_{0}$	1.83
pLFP smoothing	$T_{smoothing}$	42.2 ms
Percentile for pLFP lower bound	$p_{0}^{thre}$	1%
pLFP lower bound	$p_{0}$	2.85 ± 0.73 μV
State window	$T_{state}$	400 ms
Sliding mean window	$T_{mean}$	500 ms
pLFP threshold for state validation	$p_{fluct}^{thre}$= $p_{0}$	2.85 ± 0.73 μV
factor for rhythmicity threshold	α	2.87

**Figure 3.**
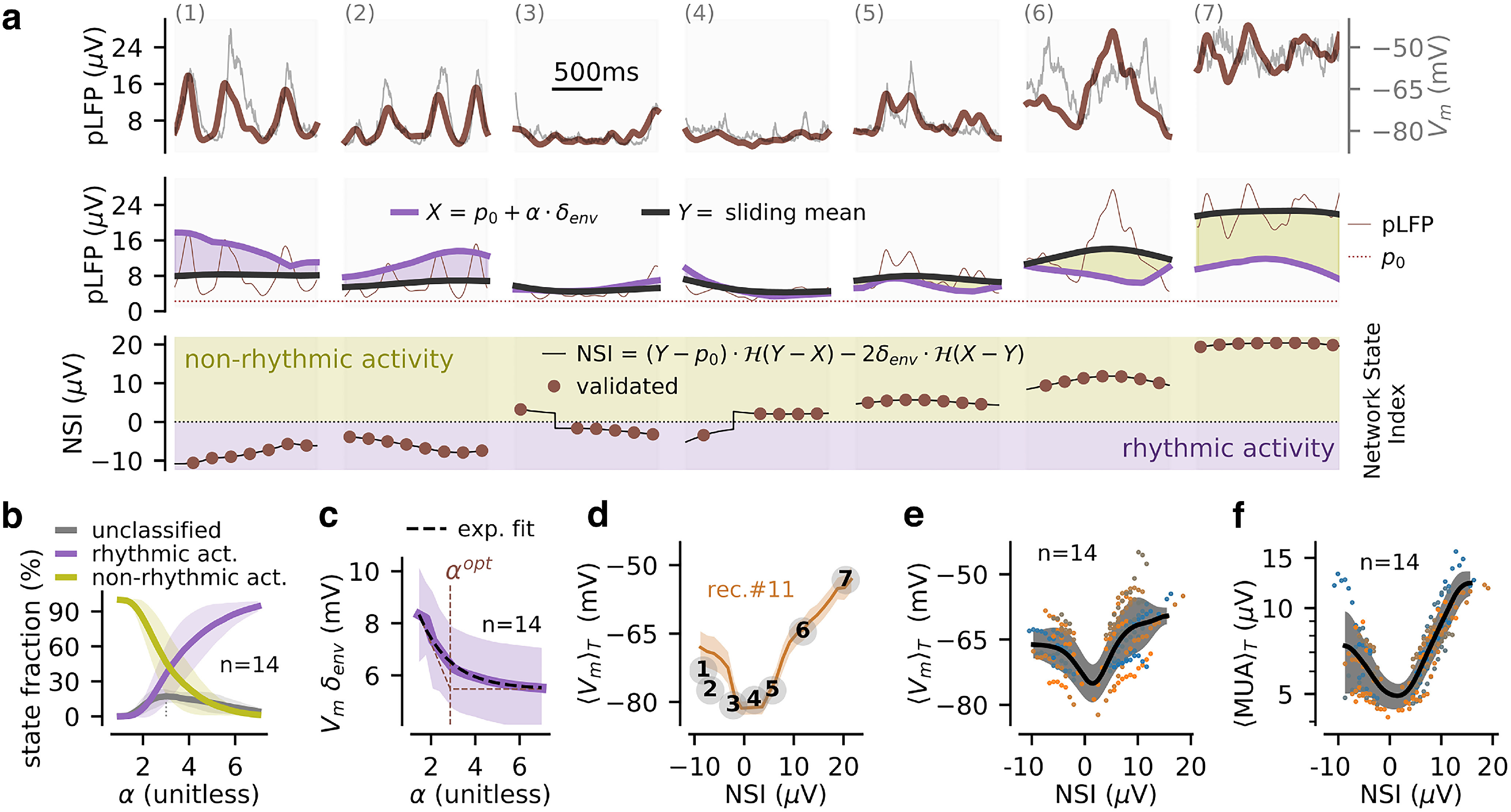
The Network State Index based on the processed LFP (NSI_pLFP_). We define a graded measure of network states based on the mean level (for nonrhythmic activity) or the low frequency envelope (for rhythmic activity) of the time-varying pLFP signal. ***a***, Example epochs of activity at different NSI_pLFP_ levels (see bottom plot), the epochs are identical to those of Figure 1*b* (recording #11). In the top plot, we superimpose the pLFP fluctuations and the V_m_ fluctuations. In the middle plot, we illustrate the signal processing steps leading to the NSI_pLFP_ measure (see main text and Materials and Methods). Two time-varying quantities derived from the pLFP signal are used to classify network states: a weighted estimate of the low frequency content of the pLFP signal X(t) (purple line, shown for α = 2.87) and the pLFP sliding mean Y(t) (black line). A consistency criterion validates a fraction of those as “validated” network states (brown dots). ***b***, Fraction of rhythmic, nonrhythmic and unclassified states as a function of the parameter α (weighting the propensity to classify as rhythmic states). ***c***, V_m_ envelope of the [2,4] Hz band averaged across all identified rhythmic states (mean ± SEM over the *n* = 14 recordings) for different values of the parameter α. We fit the decay with an exponential function (dashed red line) and take its decay parameter as the optimal value α^opt^ for the classification. ***d***, Mean depolarization level (shown as mean ± SEM over episodes at a given NSI_pLFP_ level) as a function of the NSI_pLFP_ measure for a single recording (recording #11). We highlight how the NSI_pLFP_ measure classifies the episodes shown in ***a*** (see main text). ***e***, Mean depolarization level as a function of NSI_pLFP_ for the *n* = 14 recordings of the dataset. We show the mean relation per recording (color-coded dots) and the mean and variability over recordings after Gaussian smoothing of 5 μV width (black curve and gray area, respectively). ***f***, Relationship between MUA (see Materials and Methods) and NSI_pLFP_ over episodes for all recordings (color-code following ***e***).

**Figure 4.**
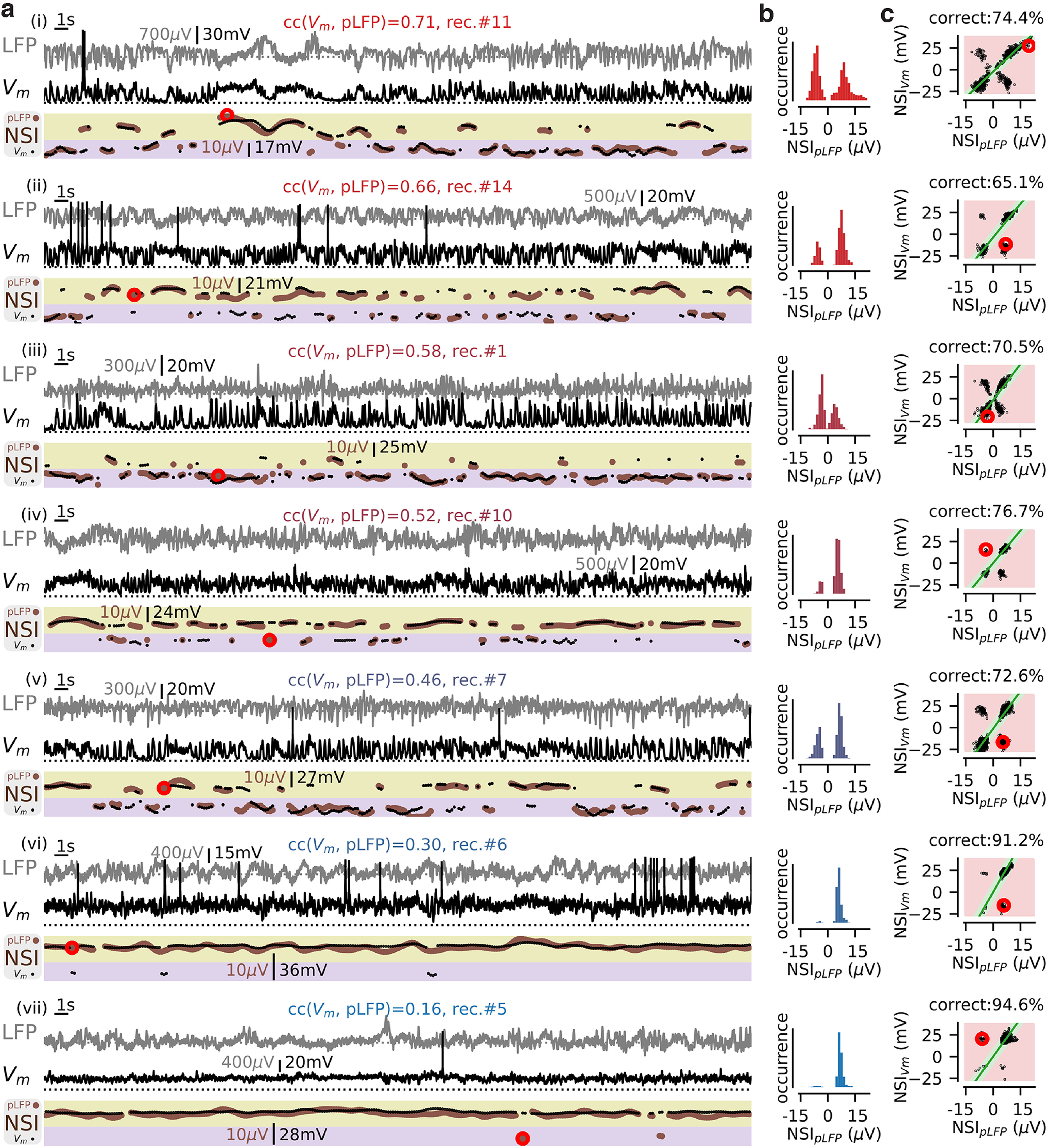
The pLFP-based NSI across multiple recordings: variability and estimated accuracy. We show seven recordings (from i to vii) covering the whole range of observed values of correlations between the intracellular (V_m_) and extracellular (pLFP) signals. ***a***, A 60-s sample of the simultaneous LFP (gray) and V_m_ (black) signals. At the bottom, we show the validated network states with their NSI value both for the “pLFP-defined NSI” (NSI_pLFP_, brown dots) and “V_m_-defined NSI” (NSI_Vm_, black dots). ***b***, Histogram of the NSI_pLFP_ over the whole recording length for each recording. The color code per recording (from red to blue, see also Fig. 2*c*) represents the value of the correlation coefficient between the V_m_ and pLFP signals. ***c***, Scatter plots of the “V_m_-defined NSI” (NSI_Vm_) and the “pLFP-defined NSI” (NSI_pLFP_) values for all validated episodes in a given recording (i.e., extending before and after the recording sample shown in ***a***). We highlight the correct area with a green color and the incorrect area with a red color. The large red circles give examples of the different sort of rejections that may happen during cross-validation (see main text). See Extended Data Figure 4-1 for the accuracy estimate with different dataset segmentation.

**Figure 5.**
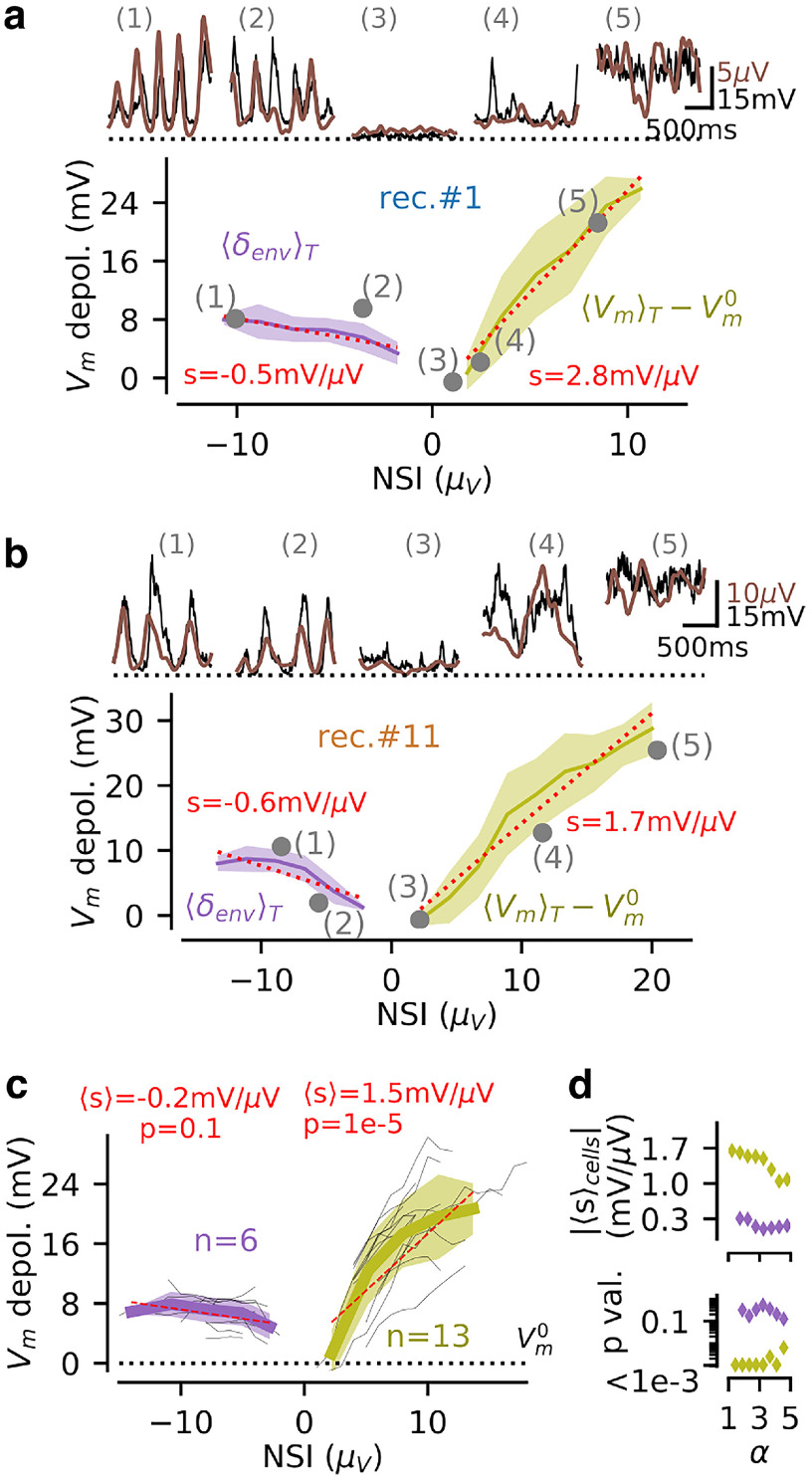
Relationship between pLFP-derived population activity (NSI_pLFP_) and single-cell depolarization (V_m_) in rhythmic and nonrhythmic regimes. ***a***, Relationship, at the single recording level (shown for recording #1), between the NSI_pLFP_ levels and the properties of the V_m_ fluctuations. For rhythmic states (NSI_pLFP_ ≤ 0, purple color), we show the relationship between the NSI level and the V_m_ amplitude of the δ-band envelope. For nonrhythmic states (NSI_pLFP_ > 0, kaki color), we show the link between the NSI level and the mean *V_m_* depolarization level over a *T_state_* = 400 ms window. We highlight with gray dots the values of the single episodes visible on Figure 3*a* (reproduced on the top inset, V_m_ in black and pLFP in brown). We show the linear regressions for the rhythmic and nonrhythmic data (dashed red line). Note that the plain curve does not reach episode 3 because the minimum number of episodes for averaging is not reached at that level (see Materials and Methods). ***b***, Same than **a** for recording #11. ***c***, Reproducing the analysis of ***a***, ***b*** over all *n* = 14 recordings (see main text). We show the mean relations over recordings (thin gray lines) and the mean (wide curve) and SD (shaded area) across recordings. Evaluated only for the NSI_pLFP_ levels displayed by multiple recordings (i.e., *n* = 6 recordings for rhythmic activity and *n* = 13 recordings for nonrhythmic activity). For each recording, we perform a linear regression with respect to the NSI_pLFP_ levels, we compute the mean across recordings 〈s〉 and the probability of a deviation from the 0-slope hypothesis *p* (paired *t* test). ***d***, Relationship between the α parameter (that sets the proportion of nonrhythmic episodes; see Fig. 3*c*) and the mean slope over cells (top, 〈s〉_cells_) together with the *p*-value testing the significance of a non-zero slope (bottom), i.e., reproducing the analysis of ***c*** for different α values.

**Figure 6.**
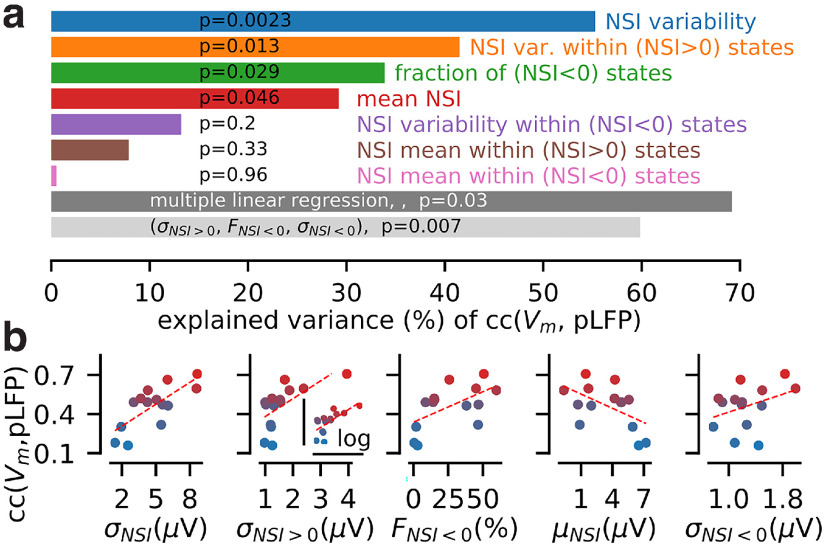
Features in the distribution of the pLFP-based NSI explain the diversity over recordings of the correlation value between the population (pLFP) and single-cell (V_m_) signals. ***a***, We perform a linear-regression between the correlation coefficient cc(V_m_,pLFP) and the following quantities characterizing the NSI_pLFP_ distribution: (1) the variability σ_NSI_ (SD) of the NSI_pLFP_ values across the whole recording, (2) the variability in NSI_pLFP_ values restricted to nonrhythmic episodes σ_NSI>0_ , (3) the fraction of rhythmic episodes F_NSI≤0_ , (4) the mean NSI_pLFP_ over the whole recording μ_NSI_, (5) the variability in NSI_pLFP_ values restricted to rhythmic episodes σ_NSI≤0_ , (6) the mean in NSI_pLFP_ values restricted to nonrhythmic episodes μ_NSI>0_ , (7) the mean in NSI values restricted to rhythmic episodes μ_NSI≤0_. We show the explained variance for all quantities on the *x*-axis and the statistical significance of those linear models (*p*-values). We also performed multiple linear regressions with all those factors (dark gray bar) and we show the best three component model (light gray bar: σ_NSI>0,_ F_NSI≤0,_σ_NSI≤0_). We report the variance corrected by the number of linear factors (adjusted *R*^2^). ***b***, Scatter plot between the value of a given factor and the V_m_-pLFP correlation coefficient across individual recordings. Shown for the first five factors of the NSI_pLFP_ distribution with the highest percentage of explained variance (see variance explained and *p*-values in ***a***). The color code of individual recordings matches that of Figure 2*c*.

Values used for the S1 dataset (Figs. 2-6). Note that the $p_{0}$ and $p_{fluct}^{thre}$ parameters are data-driven quantities, i.e., varying from recording to recording, deriving from the value of $p_{0}^{thre}$ (mean ± SEM over the *n* = 14 recordings).

### Computing the Network State Index (NSI)

Then, from the pLFP we computed the NSI, as follows. The pLFP was first downsampled by averaging over bins of 1 ms (keeping the 50 kHz sampling of electrophysiological signals is unnecessary given the much slower time scale of state transitions, see Results). We then computed the distribution of the pLFP over the whole recording and we extract the baseline noise level of the pLFP signal $p_{0}$ by taking the value of the first (lowest) percentile of the distribution. We interpret this recording-specific $p_{0}$ value as the residual high-γ envelope in absence of neural activity (see the systematic depolarization from the activity at pLFP ≤ p_0_ in Fig. 2*a*) and we therefore considered it as an estimate of the noise level in the pLFP signal.

We computed the time-varying envelope of the [2,4] Hz band $\delta_{env}$ of the pLFP signal using the above wavelet transform (Eq. 2). We took a set of 20 wavelets uniformly sampling the [2,4] Hz band and, at every 1-ms time point, we extract the maximum envelope from this band. We constructed a weighted estimate X(t) of the low-frequency content of the pLFP signal by $X\left(t\right)=p_{0}+\alpha \cdot \delta_{env}$, where α is the threshold parameter for the rhythmic/nonrhythmic classification (see Results). We also computed a slow average of the pLFP fluctuations with a Gaussian smoothing of time constant $T_{mean}$ = 500 ms, yielding the signal Y(t).

Finally, the NSI was defined from the above computed signals by the following equation:

(4) $$NSI\left(t\right)=-2\cdot \delta_{env}\cdot H\left(X-Y\right)+\left(Y-p_{0}\right)\cdot H\left(Y-X\right),$$

where H is the Heaviside step function.

**Figure 7.**
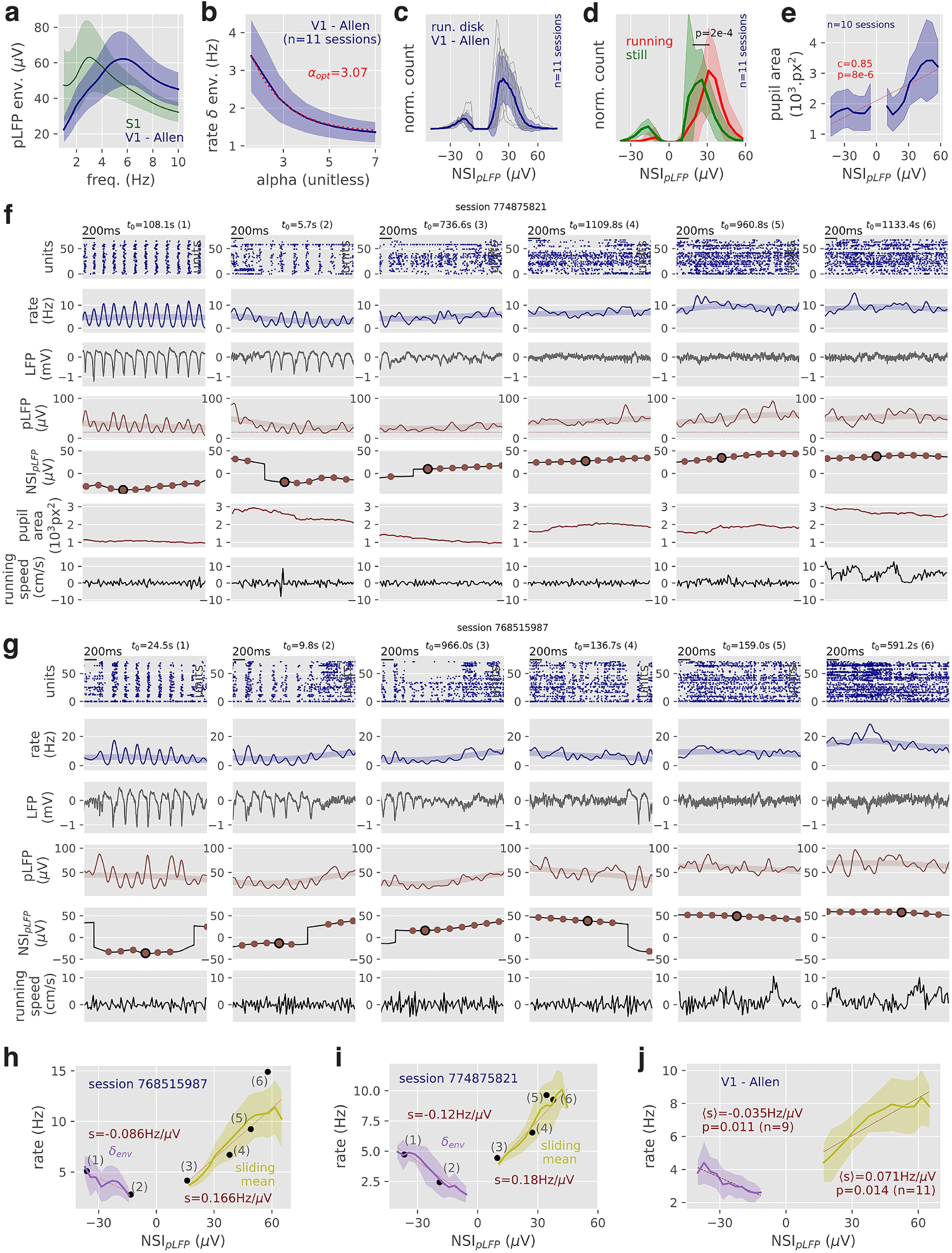
Application of the NSI analysis to the “Visual coding – Neuropixels” dataset of the Allen Institute (Siegle et al., 2021). ***a***, pLFP envelope in the δ range for the Allen dataset in V1 and in our S1 dataset for comparison. ***b***, Mean δ envelope in the spiking rate (Materials and Methods) across all pLFP-defined rhythmic episodes as a function of the α parameter. ***c***, Network state variability in the Allen dataset captured by the pLFP-defined NSI. Thin lines represent single session data. ***d***, Histogram of pLFP-defined NSI values depending on running conditions. The “running” condition corresponds to episodes when the absolute speed is >5cm/s and “still” when the absolute speed is <5 cm/s. ***e***, Relationship between pLFP-defined NSI levels and average pupil area at those levels. ***f***, Example episodes (from left to right) in session 774875821. From top to bottom, The single unit spikes over time, the time-varying rate computed from those spikes (thick transparent line: sliding mean), the raw LFP in the selected channel (see Materials and Methods), the pLFP signal (thick transparent line: sliding mean), and the pLFP-defined NSI with the validated episodes (validated episodes as dots). ***g***, Same as in **f** for session 768515987. ***h***, Relationship, at the single session level, between the NSI_pLFP_ levels and the rate fluctuations (shown as mean ± SEM over episodes). For rhythmic states (NSI_pLFP_ ≤ 0, purple color), we show the relationship between the NSI level and the rate amplitude of the δ-band. For nonrhythmic states (NSI_pLFP_ > 0, kaki color), we show the link between the NSI level and the mean rate level over a *T_state_* = 400 ms window. We highlight with black dots the values of the single episodes shown in ***g***. We show the linear regressions for the rhythmic and nonrhythmic data (dashed red line). ***i***, Same as in ***h*** for session 774875821. ***j***, Reproducing the analysis of ***h***, ***i*** over all *n* = 11 sessions. Evaluated only for the NSI_pLFP_ levels displayed by multiple recordings (i.e., *n* = 9 recordings for rhythmic activity and *n* = 11 recordings for nonrhythmic activity; Materials and Methods). For each recording, we performed a linear regression with respect to the NSI_pLFP_ levels, we computed the mean across recordings 〈s〉 and the probability of a deviation from the 0-slope hypothesis *p* (paired *t* test). In ***a,b,c,d,e,j***, data are shown as mean ± SEM (thick line with shaded area) over the sessions of the dataset.

From this time-varying signal, we computed what we termed “validated” network states by running through the time axis in steps of $T_{state}$ /2 = 200 ms and identifying those time periods in which the $T_{state}$ = 400 ms window surrounding each time point does not contain variations of the NSI signal larger than the noise level $p_{0}$. When averaging quantities for a given NSI level (Figs. 3-5, 7), we considered only the NSI levels including more than five validated episodes to get a meaningful average (thus discarding a few recordings in the population analysis because of a too low number of rhythmic episodes; see figure legends).

The signal processing steps of this procedure are illustrated on Figure 3*a*, and all parameters of the analysis are summarized in Table 1 for our dataset.

### Analysis of the “Visual coding – Neuropixels” dataset

We analyzed data from the publicly available Allen Institute for Brain Science Brain Observatory data (Siegle et al., 2021). The full data collection methodology as well as the download instructions can be found at the link https://allensdk.readthedocs.io/en/latest/visual_coding_neuropixels.html. We restricted the analysis to recordings in primary visual cortex (V1) of wild-type mice in absence of visual stimulation (i.e., focusing on the 20 min in the center of the grey screen presentation episode in the “functional connectivity” recordings). These constraints resulted in a collection of recordings from *n* = 11 mice. After selecting the probe located in V1, we defined the LFP signal by choosing a single channel within all available channels in the V1 column (∼20 channels per Neuropixels probes). We selected the LFP channel with the strongest mean pLFP envelope in the δ range over the whole session. By pooling all the available well-isolated single-unit spikes of the V1 channels, we computed a time-varying population rate by binning spikes in bins of 5 ms and smoothing the resulting trace with a Gaussian smoothing of width 30 ms. We also extracted the running speed and pupil area (π·width·height after their ellipse fit) from the dataset. All modalities (rate, running speed, pupil) were resampled to the LFP sampling using nearest-neighbour interpolation during the analysis.

### Statistical analysis

Experimental data were imported in Python using the neo module (Garcia et al., 2014). All signal processing steps (subsampling, convolution, filtering) were implemented in numpy (Harris et al., 2020). Statistical analysis was performed with the *scipy.stats* module of SciPy (Oliphant, 2007). We analyzed the linear relationship between continuous samples with a Pearson correlation analysis (function *scipy.stats.pearsonr*), and we reported the two-tailed *p*-value of the null correlation hypothesis. For the statistics of samples consisting of an averages over a given recording session (*n* = 14 recordings sessions), we tested the significance using two-tailed *t* tests (functions *scipy.stats.ttest_rel*, *scipy.stats.ttest_ind*, or *scipy.stats.ttest_1samp* for paired samples, unpaired samples and single samples respectively). The multiple linear regressions of Figure 6 were performed with the OLS (ordinary least squares) function of the *statsmodel* module. We analyzed the statistical significance of the single or multicomponent models with an *F* test, and we report the variance adjusted by the numbers of factors.

### Software accessibility

We implemented the described analysis into a software publicly available at the following link https://github.com/yzerlaut/Network_State_Index (Zenodo archive: https://doi.org/10.5281/zenodo.6597313).

## Results

### Simultaneous intracellular and extracellular dynamics in the somatosensory cortex of awake mice: variability and network states of wakefulness

We performed simultaneous closely-located (∼200–250 μm) recordings of the LFP and the membrane potential (V_m_) of pyramidal cells (see traces from two example recordings in Fig. 1*a*) in the superficial layers of the barrel cortex (S1). Recordings were performed in awake head-fixed mice habituated to sit quietly on the recording rig (Materials and Methods; see also Poulet and Petersen, 2008). Recordings had a duration of 5.1 ± 2.0 min (*n* = 14 from 4 mice). Before proceeding to use these data to define indices of network states from the LFP, we document some of its basic properties.

First, we found a notable heterogeneity across recordings (compare recording #2 with #11 in Fig. 1*a*). In particular, the relationship between extracellular population (LFP) and intracellular (V_m_) signals was highly variable (Fig 1*c*, recordings were sorted by their level of absolute correlation, note the large range of observed correlation values) and this variability could not be explained by cellular properties of the recorded cell (such as the membrane resistance and action potential threshold; see Extended Data Fig. 1-1). Second, intracellular and extracellular dynamics had a rich repertoire of activity patterns (illustrated in Fig. 1*b*). As previously reported under similar conditions (Poulet and Petersen, 2008; McGinley et al., 2015a; Vinck et al., 2015; Chen et al., 2017; Einstein et al., 2017), both LFP and V_m_ traces displayed epochs of rhythmic activity in the δ-band (defined as time samples with high [2,4] Hz envelope, see the V_m_ and LFP spectrums in Fig. 1*d*,*h*, respectively, peaks were observed at 3.0 ± 0.3 Hz for the V_m_ and 3.3 ± 0.5 for the LFP, *n* = 14 recordings). Those rhythmic epochs presented a high synchronization between LFP and V_m_ (correlation between V_m_ and LFP δ envelopes across time samples: 0.55 ± 0.11, one-sample *t* test for positive correlation, *p* = 2e-10, *n* = 14). Examples epochs of rhythmic activity are shown in traces number 1 and 2 of recording #11 (Fig. 1*b*). Next, confirming previous observations (McGinley et al., 2015a; Neske et al., 2019; Zerlaut et al. 2019; Nestvogel and McCormick, 2022), we found nonrhythmic epochs (here defined as time samples with a V_m_ δ envelope lower than 6 mV; see Fig. 1*e*) at different depolarization levels (see the population histogram on Fig. 1*f* ranging from ∼−80 mV hyperpolarization levels to ∼−45 mV depolarization levels). Example epochs of nonrhythmic activity patterns at different depolarization levels (increasing from epoch 3 to 7) are shown in Figure 1*b*. Taken together, the epochs 1–7 of Figure 1*b* recapitulate the different states described by the “U-model” of cortical states (McGinley et al., 2015b), that is rhythmic states of δ-band activity and nonrhythmic states at various membrane potential depolarization levels (from hyperpolarized to depolarized). Finally, recordings also differed not only in terms of the average strength of δ rhythmicity, but also in terms of the distribution of V_m_ levels over time (Fig. 1*f*). Recordings with lower average δ envelope tended to have lower variability of V_m_ levels over time in nonrhythmic states (Fig. 1*g*, Pearson correlation between mean V_m_ δ envelope per recording and V_m_ SD of nonrhythmic time samples, c = 0.83, *p* = 2e-4). The diversity of recordings thus filled a continuum between two qualitatively different cases of either recordings displaying mostly nonrhythmic states at an almost constant depolarization level (e.g., recording #2 in Fig. 1*a*, see V_m_ histogram in Fig. 1*f* showing a low variability of mean V_m_ over time) and recordings exhibiting overall stronger time-averaged δ V_m_ envelope and a much wider variation of the V_m_ depolarization levels (e.g., recording #11 in Fig. 1*a*, see V_m_ histogram on Fig. 1*f*). These latter cases exhibited a complex dynamics with the alternation of oscillatory δ-band activity together with nonrhythmic activity at very different V_m_ depolarization levels (see example epochs of recording #11 in Fig. 1*b*). In the next sections, we investigate how to quantitatively classify and differentiate these network states based on the extracellular LFP.

### Limitations of the existing spectral analysis of LFP for the characterization of network states with different degrees of membrane potential rhythmicity and depolarization

Because of the high impact of different strength of V_m_ rhythmicity and V_m_ depolarization levels on behavior and sensory function, we next considered how to determine a quantitative index of networks states with such V_m_ features from the more easily accessible LFP signal. Ideal properties of this index would include: (1) the ability to predict the rhythmicity and depolarization of V_m_ from the LFP; (2) a U-shaped dependence of the index on depolarization levels of membrane potential and of firing of local neural populations to directly map onto the U-model of network states.

We first evaluated whether existing methods based on simple spectral properties, could be used to characterize in this way, using only the LFP, the diversity of network states observed in the awake neocortex. Previous LFP-based characterization of different network states relied on the ratio between δ and γ power (Cheng-yu et al., 2009; Saleem et al., 2010). We therefore computed the time-varying δ [2,4] Hz and γ [30,80] Hz envelope of the LFP, and the γ-to-δ envelope ratio over time samples (see example single recording and population histograms in Fig. 1*i*). We investigated the ability of the γ-to-δ ratio to differentiate between epochs of activity that have dynamical features resembling the network states previously documented with V_m_ and described by the U-model of cortical states (McGinley et al., 2015b). To gain intuition, we first considered the example epochs 1–7, which were sorted according to the γ-to-δ ratio of their LFP (Fig. 1*i*, see the corresponding time-varying δ and γ envelopes for those epochs in Fig. 1*b*). While this ratio could distinguish the strongly rhythmic epochs (1,2) from the high-γ and highly depolarized nonrhythmic epoch 7 (Fig. 1*i*), it could not distinguish well different V_m_ depolarization levels within the nonrhythmic sets of epochs. Epoch 6 had a mean V_m_ depolarization value >15 mV higher than that of epochs 3, 5, but all these three epochs had similar γ-to-δ ratios (Fig. 1*i*). Moreover, a nonrhythmic epoch (4) had similar γ-to-δ ratio to the two rhythmic epochs (1, 2). Overall, when quantifying the dependence of mean depolarization level on the γ-to-δ ratio across all epochs for either the example recording #11 (Fig. 1*j*) or across all sessions (Fig. 1*k*), it was apparent that, using the γ-to-δ LFP ratio, it would be very difficult to distinguish between rhythmic states and nonrhythmic hyperpolarized states, and to distinguish between hyperpolarized and depolarized states within the nonrhythmic range. Next, because states with nonrhythmic and hyperpolarized V_m_ are accompanied by a reduced level of spiking activity (McGinley et al., 2015a; Neske et al., 2019; Zerlaut et al., 2019; Nestvogel and McCormick, 2022), we also analyzed the level of population firing by computing the MUA from the extracellular recordings (see Materials and Methods). Similar to what we observed with the average V_m_ depolarization (Fig. 1*k*), we found that the γ-to-δ LFP ratio had a very weak predictive power with regard to the population spiking activity (Fig. 1*l*). Thus, the γ-to-δ ratio could not be used to identify states of reduced spiking network activity during nonrhythmic activity. Reduced depolarization levels and spiking activity are important as they identify specific network states which are characterized by different properties of sensory information processing during wakefulness (Reimer et al., 2014; McGinley et al., 2015a; Vinck et al., 2015; Neske et al., 2019).

We concluded that the γ-to-δ LFP ratio poorly differentiated rhythmic and nonrhythmic states and, more importantly, did not enable to evidence different levels of depolarization within the nonrhythmic states observed in cortical dynamics under awake condition. In the next section, we introduce a processing step of the LFP that allows such characterization.

### Using the time-varying high-γ envelope of the LFP for a richer network state characterization

Because the membrane potential V_m_ is the reference signal for cortical state classification (Poulet and Petersen, 2008; Polack et al., 2013; Reimer et al., 2014; McGinley et al., 2015a; Einstein et al., 2017; Arroyo et al., 2018), and because we wanted to capture from the LFP the finer features of V_m_ dynamics (including the variations in rhythmicity and depolarization levels posited by the U model) that cannot be captured by the simple δ-to-γ ratio, we next investigated whether simple mathematical transformations of the LFP displayed temporal fluctuations more qualitatively similar to those of the membrane potential.

The inverted LFP (–LFP) displays high correlation values (cc ∼0.5) with the membrane potential in awake animals (Poulet and Petersen, 2008; Arroyo et al., 2018) and could thus potentially provide a basis signal for the characterization of network states. However, the amplitude of the LFP is strongly dependent on the depth of the recording (Sakata and Harris, 2009; Kajikawa and Schroeder, 2011; Lindén et al., 2011; Smith et al., 2012; Herreras et al., 2015) and is subjected to drifts over short (<10 s, Fig. 2*a*; epochs i and ii) and long (>1 min) time scales. These factors limit the similarity between V_m_ and –LFP, and they were shown in previous work to prevent robust classification of network state during slow wave (<1 Hz) activity (Mukovski et al., 2007).

Guided by the previous findings in anesthetized animals (Mukovski et al., 2007), we hypothesized that the high-frequency content (*f* > 40 Hz, including the γ band activity) of the LFP would provide a good predictor of the depolarization level V_m_. We therefore applied a wavelet transform to the extracellular LFP and identified the frequency band maximizing the cross-correlation with the simultaneously recorded membrane potential in our dataset (Fig. 2*b*). This was performed by independently varying a root frequency *f* and a width factor *w*, yielding the frequency band [*f/w*, *f·w*]. For each frequency band, we divided the band into 20 evenly spaced wavelet frequencies, we computed the mean over frequencies of the wavelet envelope of the LFP (resulting in the time-varying envelope shown in Fig. 2*a*), and we analyzed the correlation between this transformed LFP trace and the V_m_ trace after averaging over recordings (see the individual values per recording in Fig. 2*c* sorted by recording index in the inset). We found that the band maximizing this correlation was achieved for *f_opt_* = 72.8 Hz and *w_opt_* = 1.83, i.e., the [39.7,133.6] Hz band (see Fig. 2*b*). We also found that a temporal smoothing of the LFP envelope (with an optimal value *T_opt_* = 42.2 ms; see Fig. 2*d*) enhanced its correlation with the *V_m_* signal. We refer in the following to such smoothed high-γ envelope as the pLFP (in analogy with the terminology by Mukovski et al., 2007). Following previous literature, we interpreted this quantity as an approximation to the time-varying recruitment of synaptic activity from a local region (diameter: ∼100–200 μm) surrounding the extracellular electrode (Ray et al., 2008; Katzner et al., 2009; Lindén et al., 2011; Mazzoni et al., 2011; Ray and Maunsell, 2011; Buzsáki et al., 2012; Gaucher et al., 2012; Einevoll et al., 2013).

After this transformation of the LFP, we observed a qualitative match between the previously reported V_m_ signatures of network states (McGinley et al., 2015b) and specific features of the pLFP signal. We illustrate this finding on the recording shown in Figure 3*a*. We observed rhythmic activity at different envelope levels (example epochs #1, #2) as well as nonrhythmic fluctuations at various mean levels of pLFP signal (example epochs #3–7). This similarity encouraged us to develop a quantitative NSI based on the pLFP.

### Designing a NSI from the pLFP

To better discriminate specific network states of wakefulness from the LFP, we thus developed a quantitative index from the pLFP: the pLFP-based NSI (NSI_pLFP_). The rationale and procedure to compute the NSI from the pLFP are described in the following text and is sketched graphically with example data in Figure 3*a*.

To capture the slow fluctuations of network activity over time, we computed the sliding mean *Y(t)* of the pLFP over a slow time scale (*T_mean_* = 500 ms). Because the pLFP signal had a nonzero value at all points, we quantified the baseline of the raw pLFP signal p_0_ (set as the lowest 100th percentile of the pLFP distribution, and a measure of the level of baseline noise in the extracellular signal). We then analyzed pLFP fluctuations relative to this baseline level. We classified the pLFP fluctuations at such slow time scale as either rhythmic or nonrhythmic. Rhythmicity was quantified by the time-varying low-frequency envelope of the pLFP fluctuations δ_env_(t) using a wavelet transform. We observed that establishing the rhythmic condition by only thresholding δ_env_(t) would be misleading. Indeed, the δ-band envelope was strongly co-modulated by the mean activity level Y(t) even for nonrhythmic epochs (correlation coefficients between Y(t) and δ_env_(t) in the nonrhythmic epochs defined as NSI_pLFP_ > 0: c = 0.39 ± 0.16 across the *n* = 14 recordings, significance of a positive correlation: *p* = 3.3e-10, one-sample *t* test). This indicated that the δ envelope could reach high values, and cross an arbitrary threshold in absence of strong rhythmicity. This phenomenon is visible on Figure 3*a*: the envelope in epoch 7 is equivalent to the envelope in epoch 2 without exhibiting the clear rhythmicity in the pLFP or in the V_m_ signal that characterized epoch 2 (middle and top plots, respectively). We therefore introduced a simple model-based criterion for evaluating rhythmicity based on the following reasoning. In a noiseless, purely rhythmic setting defined by pLFP(t) = p_0_ + δ_env_ · (1 + sin(2·π·f_δ_·t)), where δ_env_ is the envelope of the oscillation, we have Y(t) = p_0_ +δ _env_. If Y(t) has an additional nonrhythmic component, we get Y(t) > p_0_ + δ_env_(t) (i.e., the oscillation alone does not account for the mean level of the signal). We adapted this last relation to build our rhythmicity criterion: a higher signal mean Y(t) than the mean expected from the δ component implies nonrhythmicity. Given the nonideal nature of the signal and to compensate for the misestimation of rhythmicity in fluctuating regimes, we rescaled the slow oscillation with a parameter α (see the next section for the determination of α), i.e., we introduced the time-varying quantity X(t) = p_0_ + α·δ_env_(t). We compared the estimate of the rhythmic contribution, X(t), with the mean activity, Y(t), to quantify rhythmicity: if X(t) ≥ Y(t) activity was set as “rhythmic” (because the slow oscillation pattern is able to account for the mean activity level). Activity was defined as “nonrhythmic” otherwise. Finally, we quantified the amount of the pLFP activity in the two regimes. In the rhythmic regime, the amount of network activity was captured by the amplitude of the oscillation, 2·δ_env_(t). In the nonrhythmic regime, the pLFP deviations from baseline Y(t)-p_0_ estimated the level of ongoing activity.

The pLFP-derived NSI was defined as the amount of pLFP activity projected on the negative and positive axis for the rhythmic and nonrhythmic regimes respectively. Such a definition resulted in a continuous index where states with stronger δ components had negative values while nonrhythmic states with stronger high-γ components had higher positive values (Fig. 3*a*). As highlighted by the colored area (Fig. 3*a*, bottom plot, purple and kaki colors for rhythmic and nonrhythmic epochs, respectively), the classification into the two states relied on the sign of the difference between the X(t) and Y(t) signals (Fig. 3*a*, middle plot) followed by a projection on either the negative part of the axis weighted by the oscillation amplitude 2·δ_env_(t) for rhythmic epochs, or the positive part of the axis weighted by the increase from baseline Y(t)-p_0_ nonrhythmic epochs (Fig 3*a*, bottom plot). The described procedure for the computation of the NSI is formalized in Equation 4 in Materials and Methods.

As the time-varying signal NSI(t) might exhibit fluctuations due to noise in both the Y(t) and X(t) quantities (X and Y are derived from the noisy LFP signal and their difference might amplify noise, see for example the signal jumps in epochs 2, 3 in Fig. 3*a*), we added a consistency criterion to NSI(t) to obtain a robust state classification of individual epochs. We first defined network state “episodes” with a window of T_state_ = 400 ms and an update every T_state_/2 = 200 ms. The motivation behind the choice of this time scale T_state_ was that it offered a good compromise between two constraints: T_state_ was long enough to get well defined states (e.g., more than half a cycle for oscillations in the [2,4] Hz range) and it was short enough to catch the fast and frequent switches of network states during wakefulness (McGinley et al., 2015b). The consistency criterion for episode classification required that, within a given time window of duration T_state_, the fluctuations of the NSI signal remained within a fluctuation threshold, equal to the pLFP noise level $p_{0}$ (because this noise level provided an estimate of the amount of signal below which a variation is not a robust signal variation). If this stability condition was met, a network state in this time window was labelled as “validated.” The “validated” states are highlighted with brown dots over some sample epochs in Figure 3*a*. If this stability condition was not met, a network state at a well-defined level could not be assessed and the network state was labelled as “unclassified.” The consistency criterion prevented state validations in the presence of strong fluctuations in the time-varying NSI signal (epochs 3 and 4 in Fig. 3*a*, bottom).

### Calibration of the rhythmicity threshold in the NSI definition

The parameter α in the pLFP-based NSI weights the propensity to classify the network activity as rhythmic. Its effect is illustrated in Figure 3*b*. Increasing α increased the proportion of rhythmic states from ∼0% at α < 1 to ∼100% at α > 6. We used simultaneous V_m_ recordings to optimize α to ensure that the classification of states as rhythmic using the LFP actually finds states that would be defined as rhythmic based on the membrane potential. In Figure 3*c*, we show, as a function of α, the average across all episodes classified as rhythmic of the [2,4] Hz δ envelope of the membrane potential V_m_. The δ envelope of V_m_ of the states classified as rhythmic decreased exponentially when α increased. We thus set α to a value α^opt^ = 2.87 that was equal to the decay constant of the V_m_ envelope as a function of α. This choice ensures we detect a large enough number of rhythmic states with a genuine amount of V_m_ rhythmicity. When classifying rhythmic states using such a value in the pLFP-based NSI algorithm, we indeed obtained that the states classified as rhythmic had V_m_ δ envelope systematically larger than the states classified as nonrhythmic (paired *t* test, *p* = 1.2e-7, *n* = 14 recordings). Importantly, such a α value was found to be very close to the value maximizing the fraction of unclassified states (α = 2.95, Fig. 3*b*, thin grey dashed line), thus corresponding to the most conservative setting to classify network states.

### Electrophysiological signatures of NSI_pLFP_-defined network states

We then analyzed additional electrophysiological features of network regimes defined by the NSI_pLFP_ measure. We show the relationship between the NSI_pLFP_ level and the mean V_m_ depolarization value for a single recording in Figure 3*d* and across all recordings in Figure 3*e*. In Figure 3*f*, we show the relationship between the NSI_pLFP_ level and the MUA across recordings.

States of robust rhythmicity (high δ envelope, e.g., epoch 1 in Fig. 3*a*) showed strongly negative NSI_pLFP_ values and were associated to intermediate depolarization and MUA levels (see population data in Fig. 3*e*,*f*). When the rhythmicity was not present (low values of pLFP δ envelope, as, e.g., in epochs 3 and 4 in Fig. 3*a*), both the depolarization and the MUA levels had values close to their minimum (see population data in Fig. 3*e*,*f*). States for which the δ component did not significantly contribute to the network activity (quantified by the pLFP) were classified as nonrhythmic (i.e., NSI_pLFP_ > 0) and both their depolarization and MUA levels strongly increased with the mean level of network activity as captured by the NSI_pLFP_ (epochs 4–7 in Fig. 3*a* and population data in Fig. 3*e*,*f*). Importantly, the values of mean depolarization and of mean spiking activity in Figure 3*e*,*f* had a much lower SE for any given value of the NSI_pLFP_ than the one that was found when considering the dependence of mean depolarization and of mean spiking activity on the γ-to-δ ratio (Fig. 1*k*,*l*), suggesting that the NSI_pLFP_ is a much tighter predictor of membrane potential and of spiking dynamics than the γ-to-δ ratio of the LFP.

We conclude that the NSI_pLFP_, has several strengths, especially when compared to previous indices. The NSI_pLFP_ captured key features of V_m_-defined network states during wakefulness (McGinley et al., 2015b): it enabled extraction of membrane potential activity regimes ranging from δ-band activity, to asynchronous regimes at low activity levels and asynchronous regimes at high activity levels (Fig. 3*e*,*f*). The NSI_pLFP_ therefore provided a quantitative measure of network states that allowed extracting the U-shape nature of cortical states from LFP recordings previously documented with intracellular recordings (Fig. 3*e*,*f*).

### Evaluation of the pLFP-based NSI accuracy in estimating membrane potential-based features

The above considerations suggest that it should be possible to use the NSI_pLFP_, a measure only based in LFPs, to identify reasonably well states that have either rhythmic or nonrhythmic membrane potential properties, and to identify among the states with nonrhythmic membrane potentials, those that have either depolarization or hyperpolarization of membrane potential. In this section, we quantified the accuracy of such state characterization using the pLFP-based NSI.

To this aim, we first computed the NSI on the V_m_ signal. The lower bound of the signal $p_{0}$ was translated into the *V_m0_* value by taking the first percentile of the V_m_ distribution (see Figs. 2*a*, 4*a*). We computed the sliding mean and the time-varying low-frequency envelope with the same parameters as for the pLFP signal. We derived the *X(t)* and *Y(t)* and the “V_m_-defined NSI” (NSI_Vm_) according to Equation 4 (see Materials and Methods). This V_m_-defined NSI has (by construction) negative values for the states with V_m_ rhythmicity, low positive values for states with hyperpolarized nonrhythmic V_m_, and high positive values for states with depolarized nonrhythmic V_m_. Thus, comparing the value of the V_m_-defined and pLFP-defined NSI during the validated network states enables a simple quantification of how good is the pLFP-defined NSI at identifying states of rhythmic, nonrhythmic, depolarized and hyperpolarized membrane potential.

10.1523/ENEURO.0073-22.2022.f4-1 Extended Data Figure 4-1 Segmenting the dataset to perform the fitting and the accuracy estimate. ***a***, We consider different segmentation for the analysis of the dataset: we fit and we estimate the accuracy on the full dataset (grey label, as in the main text), or we consider a given fraction of the data to fit and the rest of the dataset to estimate the accuracy of the classification (see the schematic of the dataset segmentation for the different cases considered). ***b***, Mean envelope in the rhythmic states (see main text and Fig. 3*c*) as a function of the parameter for the different cases of dataset segmentation. We show the mean SEM over the *n* = 14 cells. ***c***, Estimated obtained from the fitting of an exponential decay of the curves shown in ***b*** (see Materials and Methods and Fig. 3*c*). Note the narrow range of variations of (2.7–3.1) for the different cases of variations. ***d***, Estimate of the NSI classification accuracy obtained from comparing the -defined and pLFP-defined NSI measure (see main text and Fig. 4*c*). We show the mean SEM over the *n* = 14 cells. All cases were found not to differ significantly from the accuracy reported in the main text, from left to right the different *p* = 0.12, *p* = 0.22, *p* = 0.52, *p* = 0.44 (paired student *t* test). Color code as in ***a***. Download Figure 4-1, TIF file.

Figure 4*a* shows the time course over different recording sessions of the NSI computed either on the pLFP or on V_m_. From these plots, it is apparent that the two NSI indices are remarkably well matched over the time epochs of the recordings, with the occasional presence of episodes in which the two indices were mismatched (e.g., those marked by red circles in Fig .4*a*). To assess when the pLFP-based NSI did and did not correctly predict the NSI measured on the membrane potential, we implemented the criterion displayed in Figure 4*c*. We first determined the scaling factor F between the V_m_-based NSI and the pLFP-based NSI, by performing the linear fit of the data predicting either both rhythmicity or both nonrhythmicity (i.e., on the lower left or the upper right of the plots in Fig. 4*c*). This linear relationship yielded a prediction for the NSI_Vm_ value from a NSI_pLFP_ value. Then, at a given time-point *t_i_*, the prediction was considered as correct if the difference between the predicted and observed value of NSI_Vm_ lied within a tolerance interval defined by two free parameters, $p^{tol}$ and $V_{m}^{tol}$. We set the value of $p^{tol}$ as the average noise level of the pLFP signal across recordings, i.e., $p_{noise}$= 2.85 μV. For the tolerance value $V_{m}^{tol}$, we took $V_{m}^{tol}$ = 2 mV to be well above the noise level of V_m_ recordings (∼0.1 mV) and still have a high resolution in the [0–35] mV range of observed depolarization levels (see Fig. 3*e*). Correct detection thus occurred when the following conditions were met: $F\cdot \left(NSI_{V_{m}}\left(t_{i}\right)+V_{m}^{tol}\right)<NSI_{pLFP}\left(t_{i}\right)+p^{tol}$ and $F\cdot \left(NSI_{V_{m}}\left(t_{i}\right)-V_{m}^{tol}\right)>NSI_{pLFP}\left(t_{i}\right)-p^{tol}$. The strictness of this criterion is illustrated in Figure 4*c*. States were taken as incorrect when there was a mismatch between the rhythmic versus nonrhythmic classification (as shown for cases ii, iv–vii in Fig. 4, see the nonmatching events highlighted with a red circle). Importantly, state classification was also taken as incorrect when the graded level of the rhythmicity or the nonrhythmicity was not predicted well enough (see Fig. 4, i, iii). For example, in recording #11 (Fig. 4, i), the NSI_Vm_ value did not display a high enough value to be linearly related to NSI_pLFP_ level according to the relationship F.

Using the tolerance criteria defined in the above paragraph, the accuracy of detection of a V_m_-based NSI value from the pLFP-based NSI value was 79.7 ± 10.2% (mean ± SEM over the *n* = 14 recordings and all validated episodes). Decreasing the tolerance parameter values to $p^{tol}$ = 1 μV and $V_{m}^{tol}$ = 1 mV (corresponding to extremely strict matching criteria) led to an accuracy of 57.2 ± 15.1%, meaning that, in more than half of the cases, the network state could be identified even with such remarkably high precision. We checked that accuracy was not artificially inflated by overfitting by splitting the dataset into different training and test sets. In this control analysis, we found very similar parameters and we did not find any significant differences in the measured accuracies (see Extended Data Fig. 4-1).

We noted that the misclassifications were not homogeneously distributed across different states (see Table 2). A specific set of network state misclassifications represented 65.3% of the misclassifications over the merged episodes across all recordings (i.e., 13.1% of all episodes). In this set, rhythmic activity predicted from the V_m_ (NSI_Vm_ ≤ 0) was misclassified as nonrhythmic activity from the pLFP (NSI_pLFP_ > 0). We analyze in the next section the reasons behind this finding.

**Table 2:** Misclassifications in the pLFP-based (NSI_pLFP_) versus V_m_-based (NSI_Vm_) NSI characterization

Misclassification cases	Percentage
NSI_pLFP_ > 0 and NSI_Vm_ ≤ 0	65.3%
NSI_pLFP_ ≤ 0 and NSI_Vm_ > 0	25.0%
NSI_pLFP_ > 0 and NSI_Vm_ > 0	5.4%
NSI_pLFP_ ≤ 0 and NSI_Vm_ ≤ 0	4.3%

Proportions of the misclassifications split into the rhythmic (NSI ≤ 0) and nonrhythmic (NSI > 0) cases for both the pLFP and V_m_ signals over all episodes in the *n* = 14 recordings (related to Fig. 4*c*).

### Graded aspect of network states in terms of pLFP and V_m_ fluctuations: rhythmic versus nonrhythmic states

The above analysis revealed a stronger tendency to classify network states as rhythmic from the V_m_ than from the pLFP fluctuations. This difference in classification suggested that the NSI measures revealed previously unexplored asymmetries between the characteristics of fluctuations of pLFP and V_m_ signal across different network states. We therefore analyzed more closely the correspondence between pLFP and V_m_ fluctuations for different levels of pLFP-based NSI and how this impacted our classification results.

For nonrhythmic activity (NSI_pLFP_ > 0), the level of the NSI_pLFP_ was given by the mean pLFP deflection (Y-p_0_) in the time window T_state_ = 400 ms. We thus compared the positive NSI values to the mean membrane potential depolarization level in the same window T_state_. For rhythmic activity (NSI_pLFP_ ≤ 0), the level of the NSI was proportional to the δ envelope of the pLFP. We thus compared the negative NSI values to the δ envelope in the V_m_ signal. We show the relationship between those pLFP-defined and V_m_-defined levels for two recordings in Figure 5*a*,*b* (for recording #1 and recording #11, respectively) and for the population data on Figure 5*c*.

Overall, we found (Fig. 5) that the membrane depolarization exhibited a strong dependency on the pLFP-based NSI level for nonrhythmic activity (NSI_pLFP_ > 0). We showed single episodes of increasing NSI_pLFP_ levels (numbered 3, 4, 5, 6 in Fig. 5*a*,*b*) and their respective episode averages at all NSI_pLFP_ > 0 levels for those two recordings (Fig. 5*a*,*b*, kaki curves). The ∼15 μV variability in terms of pLFP based NSI levels corresponded to a ∼30 mV variability of depolarization level with a clear monotonic relationship for those two sample recordings. This behavior was confirmed at the population level (Fig. 5*c*). We standardized the analysis across all recordings by binning the NSI_pLFP_ levels from 0 to 30 μV (a range covering all observed values) in bins of 1 μV. We found that all recordings exhibited depolarizations with a steep dependency on the NSI_pLFP_ level 1.5 ± 0.7 mV/μV, significantly deviating from the null hypothesis of a zero slope (*p* = 1.2e-5, *n* = 13 recordings, paired *t* test). On the other hand, we found that the value of pLFP-based NSI for rhythmic activity (NSI_pLFP_ ≤ 0) had a much lower impact on the membrane depolarization level (see Fig. 5). The ∼10 μV variability in terms of pLFP based NSI levels translated into a weakly modulated V_m_ oscillation with a 5 to 10 mV amplitude (Fig. 5*a*,*b*, purple curves). We highlight this weak dependency on the selected samples shown in Figure 5*a*,*b*. We extended the analysis to all recordings, after standardizing the data by binning the pLFP-based NSI levels from –30 to 0 μV. At the population level, we confirmed that the mean membrane depolarization had a weak dependency on the pLFP-based NSI level (–0.2 ± 0.3 mV μV), which was not significantly deviating from the null hypothesis of a zero slope (*p* = 0.14, over the *n* = 6 recordings displaying rhythmic activity within multiple NSI_pLFP_ bins, paired *t* test). It should be noted that the lack of graded V_m_ variations for rhythmic activity was not related to our “rhythmicity threshold” limiting the set of rhythmic samples to a potentially-biased subset. When varying the rhythmicity-factor α up to α = 5 (where the occurrence of rhythmic NSI_pLFP_ ≤ 0 states reaches ∼80%; see Fig. 3*b*), the depolarization level still showed a very weak dependency to the NSI level compared to that for nonrhythmic activity (Fig. 5*d*, top) with similar statistical significance values (see Fig. 5*d*, bottom).

We concluded that the graded levels of neural activity captured by the NSI_pLFP_ had a strong correlate in terms of membrane potential depolarization for nonrhythmic activity (NSI_pLFP_ > 0), while the various pLFP-based NSI levels of rhythmic activity (NSI_pLFP_ ≤ 0) rather corresponded to a stereotypical V_m_ oscillation with ∼5 to 10 mV amplitude.

Those observations explained the results of our cross-validation analysis (Table 2). The high precision of the classification in the jointly nonrhythmic case (“NSI_pLFP_ > 0 and NSI_Vm_ > 0” in Table 2, only 5.4% of the misclassifications) resulted from the strong relationship between the pLFP and V_m_ signals during nonrhythmic states (Fig. 5*a–c*, kaki curves). On the other hand, the existence of a few episodes showing a high envelope δ oscillation in the V_m_ with a low δ envelope in the pLFP (cases such as episode 2 in Fig. 5*a*,*b*) created an ambiguous situation for the classifier because rhythmicity was hard to establish from the pLFP signal in those episodes. The cases with mixed rhythmic/nonrhythmic predictions indeed represented 90.3% of the misclassifications (“NSI_pLFP_ > 0 and NSI_Vm_ ≤ 0” and “NSI_pLFP_ ≤ 0 and NSI_Vm_ > 0” in Table 2). In particular, predicting rhythmicity from the V_m_ and nonrhythmicity from the pLFP was the prevalent misclassification case (65.3% for the case “NSI_pLFP_ ≤ 0 and NSI_Vm_ > 0”), consistent with the stronger representation of the δ pattern in the V_m_ than in the pLFP (NSI_pLFP_ ≤ 0 range in Fig. 5*a–c*). We confirmed that such misclassification cases originated from episodes of low δ envelope in the pLFP signal: the mean δ_env_ in misclassified cases was significantly lower than in accurately classified cases (1.5 ± 0.3 vs 2.7 ± 0.5 μV, *p* = 3.3e-10 paired *t* test, in the *n* = 13 recordings displaying both conditions).

### Using the NSI_pLFP_ to quantify network state distributions in the somato-sensory cortex of awake head-fixed mice

We analyzed the NSI_pLFP_ state distribution in the whole dataset (*n* = 14 simultaneous V_m_ and LFP recordings in the somato-sensory cortex of awake head-fixed awake mice). Figure 4 shows eight example recordings representative of the dataset, spanning the full range of correlations between the pLFP and V_m_ fluctuations (see Fig. 2*c*). We show a 60-s sample of the intracellular and extracellular recordings together with the time-varying NSI_pLFP_ (Fig. 4*a*) and the histogram of the NSI_pLFP_ levels across the whole recording (Fig. 4*b*).

Overall, the dataset was dominated by nonrhythmic activity with a fraction of nonrhythmic states: $F_{NSI>0}$ = 81.1 ± 15.9%. The fraction of rhythmic activity exhibited a large variability with *n* = 3 recordings below $F_{NSI\leq 0}$ = 2% and *n* = 3 recordings above $F_{NSI\leq 0}$ = 35% (peaking at $F_{NSI\leq 0}$ = 46.8% for recording #1, see Fig. 4*b*).

The mean absolute NSI_pLFP_ value over rhythmic periods (i.e., NSI_pLFP_ ≤ 0) was 5.65 ± 1.02 μV (mean ± SEM over *n* = 14 recordings) and 6.67 ± 1.34 μV for nonrhythmic periods (i.e., NSI_pLFP_ > 0), yielding a significant increase from rhythmic to nonrhythmic states (*p* = 3.0e-5, paired *t* test). The increased amplitude in terms of pLFP signal (i.e., high-frequency content of the LFP) suggested that, as an average, synaptic activity was stronger in the nonrhythmic periods that in the rhythmic ones (see Discussion). This increased range of pLFP level was also true for the maximum level displayed by single recordings with 9.52 ± 2.25 versus 12.06 ± 3.4 μV (*p* = 9.8e-3, paired *t* test) for rhythmic and nonrhythmic activity, respectively. During nonrhythmic periods, we found a mild but significant increase of the variability (SD, from 1.29 ± 0.32 to 1.56 ± 0.71 μV, *p* = 8.9e-2, paired *t* test) and skewness of the pLFP distributions (from 0.35 ± 0.33 to 0.7 ± 0.41, *p* = 8.2e-2, paired *t* test).

### Network state variability within individual recordings predicts the average correlation between population and single-cell signals

We next analyzed whether the NSI_pLFP_ state distributions could explain the variability of the correlation cc(V_m_, pLFP) between the time-varying population signal pLFP(t) and the single-cell signal V_m_(t) in our recordings (see Fig. 4*a*,*b*). We reduced the NSI_pLFP_ distribution per recording to a few components (detailed below) and we used univariate and multivariate linear regressions to analyse how the variability of those components across recordings explained the variability of the observed correlation values.

From top to bottom in Fig. 4*b* (i.e., from high to low correlation recordings), the distribution of NSI_pLFP_ levels across recordings was quantitatively different. At the top [high correlations, cc(V_m_, pLFP) > 0.4, panels i–v], distributions were bimodal with two high peaks both in the rhythmic (NSI_pLFP_ ≤ 0) and nonrhythmic (NSI_pLFP_ > 0) areas. At the bottom [low correlations, cc(V_m_, pLFP) < 0.4, panels vi, vii], distributions were dominated by nonrhythmic episodes with rather narrow range of NSI_pLFP_ values within the (NSI_pLFP_ > 0) domain. To quantify these features within the single recording distributions, we decomposed each distribution into the following quantities: (1) the mean NSI_pLFP_ > 0 over the whole recording μ_NSI_; (2) the variability (SD) of the full NSI distribution σ_NSI_; (3) the fraction of rhythmic episodes $F_{NSI\leq 0}$; (4) the mean in NSI_pLFP_ values restricted to nonrhythmic episodes $\mu_{NSI>0}$; (5) the mean in NSI_pLFP_ values restricted to rhythmic episodes $\mu_{NSI\leq 0}$; (6) the variability in NSI_pLFP_ values restricted to nonrhythmic episodes $\sigma_{NSI>0};$ (7) the variability in NSI_pLFP_ values restricted to rhythmic episodes $\sigma_{NSI\leq 0}$.

We show the results of a linear regression analysis for each factor in Figure 6*a* (ordered by explained variance) and their relationship with the correlation value in Figure 6*b*. The factor explaining the highest percentage of the variability (55.27% of the full variability) was the SD of the NSI distribution $\sigma_{NSI}$. Strikingly, the fraction of (synchronous) rhythmic states was only the third most important factor with an explained variance of 33.89%. A more important factor was found to be the variability of network states within nonrhythmic states $\sigma_{NSI>0}$ with an explained variance of 41.5%. Recording #14 in Figure 4ii provided an example of these observations. It exhibited strong variability in terms of nonrhythmic states and relatively low occurrence of rhythmic activity (e.g., lower than recording #1). However, it still displayed high correlation coefficient [with cc( $V_{m}$, pLFP) = 0.66]. Other individual factors had weak statistical significance (*p* > 0.04; see Fig.6*a*). However, using a multiple linear regression including all factors, we found that different components of the NSI distributions had complementary contributions in shaping the average correlation *per* recording. The full linear model indeed yielded an explained variance of 69.17% (after correction by the number of linear factors, *f* test *p* = 3.1e-2). Reducing the dimensionality of the linear model (up to three components), we found that combining the significant subcomponents of the NSI variability ( $\sigma_{NSI>0}$ and $F_{NSI\leq 0}$) with the variability within rhythmic states $\sigma_{NSI\leq 0}$ produced a statistically-significant model (*f* test, *p* = 6.6e-3) predicting 59.84% of the variability (corrected by the number of factors).

We concluded that, in the present dataset, the average correlation between a single-cell signal (V_m_) and the population signal (pLFP) could be largely explained (up to ∼70%) by features of the NSI_pLFP_ distributions. State variability among all NSI_pLFP_-defined states ( $\sigma_{NSI}$) was a critical factor in determining such a variability (Fig. 6*a*, blue). We decomposed this state variability and found that the two most prominent factors are the variability within nonrhythmic states ( $\sigma_{NSI>0}$; Fig 6*a*, orange) followed by the occurrence of synchronous rhythmic activity ( $F_{NSI\leq 0}$; Fig. 6*a*, green).

### Computation of *NSI_pLFP_* indices in mouse visual cortex

Finally, we demonstrate the general applicability of the method on a publicly available dataset from the Allen Institute for Brain Science (Siegle et al., 2021). We analyzed neural activity recorded with Neuropixels probes (Jun et al., 2017) in the V1 of awake mice on a steering wheel during spontaneous activity (grey screen presentation). We extracted a V1-located LFP signal from the probes (Materials and Methods) and we computed the time-varying population firing rate summing spikes from all well-isolated single units (Materials and Methods). We first re-performed all calibration steps of the method on the new dataset. We found that, with respect to our V_m_ and LFP S1 dataset analyzed in previous figures, the δ oscillation in V1 was clearly shifted up in frequency (Fig. 7*a*, peak at 5.7 ± 0.6 Hz, *n* = 11 sessions). We therefore shifted the δ-band to [4,8] Hz, and we decreased the temporal smoothing (*T_smoothing_* = 30 ms) to avoid smoothing out the faster temporal dynamics of the δ oscillation. We next determined the optimal rhythmicity parameter α by analyzing the amount of δ envelope in rhythmic states when varying α (Fig. 7*b*, analysis as in Fig. 3*c*). We found an optimal value of α=3.07, close to the α= 2.85 value of the S1 dataset with *V_m_* recordings. Optimal parameters for the V1 dataset are summarized in Table 3.

**Table 3:** Parameters of the NSI_pLFP_ characterization in the “Visual coding – Neuropixels” dataset

Parameter	Symbol	Value
δ Band	*F*_δ_	[4,8] Hz
pLFP smoothing	$T_{smoothing}$	30 ms
pLFP lower bound	$p_{0}$	18.9 ± 3.7 μV
Factor for rhythmicity threshold	α	3.07

All other parameters are identical to Table 1. The $p_{0}$ parameter is a recording-specific parameter (reported as mean ± SEM over the *n* = 11 sessions) resulting from the setting $p_{0}^{thre}$=1%. As in Table 1, the pLFP threshold for state validation was set to $p_{fluct}^{thre}$=$p_{0}$ for each recording.

Similarly to our S1 dataset, we observed that network activity in V1 during wakefulness was characterized by a strong variability of network activity patterns, including δ oscillation and nonrhythmic episodes at various spiking activity levels (see raw data in Fig. 7*f*,*g* and histograms in Fig. 7*c*). The activity was overall dominated by nonrhythmic episodes with a percentage of 86.7 ± 3.3% of all validated episodes (see Fig. 7*c*), i.e., activity was ∼5% less rhythmic than in our dataset. State distributions were less variable across recordings than in our S1 dataset (e.g., rhythmic activity fraction only varies from a minimum of 10.2% to a maximum of 22.5%), likely due to the increased sampling durations (20 min here vs ∼5 min in our dataset). We used this dataset to analyze the relationship between NSI_pLFP_ values, behavioral state and population firing rates. If the NSI_pLFP_ index well captures the U-shaped relationship between network states and behavioral states, it should satisfy three properties: (1) high values during running; (2) increasing values with pupil size; (3) a U-shape relationship with firing rates. We examined next how well the NSI_pLFP_ index matches these expectations.

We first computed the network state distribution in “running” (mean absolute running speed during the episode larger than 5cm/s) and “still” conditions (Fig. 7*d*). As predicted based on previous studies (Niell and Stryker, 2010; Ayaz et al., 2013; Polack et al., 2013; Vinck et al., 2015), we found a significant shift of the mean network state toward the higher values of NSI_pLFP_ (mean NSI_pLFP_ in running 31.3 ± 9.6 vs 18.9 ± 4.7 μV in still conditions, *p* = 2e-4, paired *t* test, *n* = 11 sessions). Next, we found a strong and monotonic relationship between NSI and pupil area (Fig. 7*e*, Pearson correlation, c = 0.85, *p* = 8e-6) as reported in previous studies (Reimer et al., 2014; McGinley et al., 2015a), suggesting that the NSI_pLFP_ is a good index of the states expected by the U-model (McGinley et al., 2015b). Finally, we investigated the dependence of population firing rates on the NSI_pLFP_ index. We found that the relationship between pLFP and spiking rates significantly deviated from the null hypothesis of 0-slope both in the rhythmic and nonrhythmic regimes (one sample *t* test with slope values, *p* < 0.05 in both cases, see values in Fig. 7*j*), with a positive slope for positive NSI_pLFP_ values and a negative slope for negative NSI_pLFP_ values. Thus, the NSI_pLFP_ had a U shape relationship with the population firing rate.

## Discussion

In this study, we developed a method to extract from LFP recordings in the awake mouse cortex network states information that previously could be obtained only with intracellular V_m_ recordings (Poulet and Petersen, 2008; Polack et al., 2013; Reimer et al., 2014; McGinley et al., 2015a; Einstein et al., 2017; Arroyo et al., 2018; Nestvogel and McCormick, 2022). Prolonged membrane potential recordings are difficult to achieve, and most of the times require head-fixation (but see Lee et al., 2006). This strongly limits our ability to describe the complexity of network states and their relationship with behavior. Achieving precise state classification based on LFP recordings, which are technically easier to perform and can be performed in freely moving animals, will greatly increase our ability to understand the cellular and network mechanisms underlying cortical processing during behavior. Previous attempts to classify network states from the LFP have been limited to the δ-band activity (Vinck et al., 2015; Chen et al., 2017; Pala and Petersen, 2018). When applied to data gathered in awake animals, the δ-to-γ state classification used in anesthetized preparations (Cheng-yu et al., 2009; Saleem et al., 2010) was shown to only separate between the two extremes of the spectrum of cortical states: synchronized δ-band activity and desynchronized activity at high-γ power. In contrast, the new classification method developed in this study, NSI, captured the large spectrum of network states in the awake neocortex. Importantly, it provided quantitative measurement of the “U-model” of cortical states, which was previously developed only based on intracellular membrane potential recordings (McGinley et al., 2015b).

Prior to the NSI classification, we found it essential to apply a preprocessing step to the extracellular LFP. We computed the time-varying envelope after a wavelet transformation in the high-γ band (yielding the pLFP signal). In a previous study in cat neocortex (Mukovski et al., 2007), the authors identified active and silent states (up and down, respectively) under anesthesia from the pLFP signal (with a slightly different frequency band of the LFP: 20–100 Hz, instead of the data-driven [39.7,133.6] Hz band used here). We showed that such a signal processing step enabled capturing various states of wakefulness, ranging from δ (∼3 Hz) oscillatory activity to desynchronized states at various levels of spiking activity.

We then used the NSI to analyze how the distribution of network states within a recording period shaped the average correlations between the single-cell (intracellular) and population (extracellular) signals. Such diverse correlation levels seemed not to be explained by single-cell features (Extended Data Fig. 1-1) and are unlikely to result from a variable distance between electrodes across recordings (Arroyo et al., 2018), given the close and narrow variability setting of our experiments (200 to 250 μm distance between electrodes; see Materials and Methods). In anesthetized preparations, a consolidated view suggests that low-frequency activity is the main source for neural synchrony in cortical networks and thus for cell-to-population correlation (Steriade et al., 1993). Also in our dataset recorded during wakefulness, the fraction of slow (δ-band) oscillatory episodes was a factor significantly contributing to the level of correlation between single-cell and population signal (Fig. 6). However, and unexpectedly, we found that this was a weaker factor than the variability in the set of nonrhythmic states NSI values of cortical activity (Fig. 6). This observation was explained by the fact that synchronized δ activity represented only a modest fraction of network activity during wakefulness (18.9% in the S1 dataset, 13.3% in the Allen V1 dataset) and by the fact that the diverse nonrhythmic states corresponded to strongly differing levels of both the V_m_ depolarization and the high-γ activity (Reimer et al., 2014; McGinley et al., 2015a; Zerlaut et al., 2019; Figs. 2-4). Importantly, the diversity of network state distributions across recordings largely contributed to the high variability in the measured cell-to-population correlations (70% of this variability could be explained by recording-specific features of the NSI_pLFP_ distribution; Fig. 6). This observation further highlights the necessity of network-state monitoring in the interpretation of experimental results in the awake cortex (McGinley et al., 2015b), and it suggests the possible importance of NSI indices to understand and characterize how the degree of coupling between single-cell and population-level activity varies across different behavioral states.

Our results provide possible insights on the circuit dynamics during different cortical states observed during wakefulness. First, nonrhythmic episodes had population activity levels (pLFP) varying over a wide range and seemed to reach up to levels never observed in rhythmic episodes (Fig 3*f*). Second, in such nonrhythmic states, we observed a tight relationship between the population activity and single-cell depolarization (Fig. 5). This suggests that local recurrent spiking activity plays a major role is shaping the dynamics of nonrhythmic states. It also corroborates, at the level of population signals, that the hyperpolarized nonrhythmic states observed at intermediate arousal when sensory detection is optimal are characterized by lower levels of ongoing recurrent synaptic activity in local cortical populations (McGinley et al., 2015a; Neske et al., 2019; Nestvogel and McCormick, 2022). Instead, during rhythmic states, population activity varied over a much more limited range, and the relationship between population activity (pLFP) and single-cell membrane potential was much less tight (Fig. 5). This suggests that, in rhythmic states, strong depolarization patterns can be evoked despite a low level of spiking activity in the local recurrent population (see Fig. 5 vs Fig. 7). Such a phenomenon might be explained by the fact that: (1) the slow rhythmic ∼3 to 6 Hz oscillatory activity originates from thalamo-cortical interactions (Nestvogel and McCormick, 2022) and (2) the V_m_ can display high amplitude patterns in response to a few excitatory-only inputs while the LFP may only display strong signals at higher recruitment levels involving inhibition (Teleńczuk et al., 2017). Because of the occasionally low level of the pLFP signal during rhythmic activity, the NSI_pLFP_ was biased towards low activity nonrhythmic states when compared to intracellular recordings. Despite this limitation, the overall high matching value obtained through cross-validation (∼80% correct) suggested that the pLFP-based NSI is a valid index to characterize the different network states occurring during wakefulness.

Given the widespread use of extracellular recordings in neuroscientific research in both head fixed and freely moving preparations (Panzeri et al., 2015; Jun et al., 2017) and the relevance of state modulation in sensory processing (Niell and Stryker, 2010; Vijayan et al., 2010; Destexhe, 2011; Ayaz et al., 2013; Bennett et al., 2013; Ecker et al., 2014; Fu et al., 2014; Lee et al., 2014; Pinto et al., 2013; Polack et al., 2013; Reimer et al., 2014; Zhou et al., 2014; Pachitariu et al., 2015; Vinck et al., 2015; Arandia-Romero et al., 2016; Pakan et al., 2016; Busse et al., 2017; Dadarlat and Stryker, 2017; Einstein et al., 2017; Muller et al., 2018; Poulet and Crochet, 2019; Davis et al., 2020), the presented method provides a potentially important analytical tool to document the properties and functions of state-dependent computations in neocortex (Buonomano and Maass, 2009; Cardin, 2019).

## Synthesis

Reviewing Editor: Nicholas J. Priebe, University of Texas at Austin Decisions are customarily a result of the Reviewing Editor and the peer reviewers coming together and discussing their recommendations until a consensus is reached. When revisions are invited, a fact-based synthesis statement explaining their decision and outlining what is needed to prepare a revision will be listed below. The following reviewer(s) agreed to reveal their identity: NONE.

SYNTHESIS

Two reviewers evaluated the manuscript “Network States Classification based on Local Field Potential Recordings in the Awake Mouse Neocortex” and both reviewers agree that the authors provide a compelling new analytical approach that may help others in the field to identify awake network states more accurately in LFP recordings. The reviewers also agreed that a central strength of the manuscript lies in its use of whole cell recordings to estimate network state. There was consensus, both in the reviews and in the consultations that followed, that there are issues that must be addressed to make the manuscript suitable for publication. There is concern about how general this method is to recover network state. Indeed, there was some concern that using membrane potential fluctuations alone to infer network state may be problematic because of the underlying neuronal diversity. Both reviewers noted that the procedure had problems identifying transitions between rhythmic states and non-rhythmic states (Fig. 4). These issues and others are detailed below, which you should find useful in revising your manuscript, if you decide to resubmit it to eNeuro or to another journal.

Major Issues:

1) What should be the ground truth for identifying network state? In the significance statement, the authors state: “a detailed characterization of network states in the awake cortex has mostly been achieved through single-cell intracellular recordings, which however cannot be easily recorded”. This statement a little misleading since it sounds as if intracellular recordings are most often used to characterize network states in the awake brain. There is a very long history of using EEG/LFP recordings to characterize and classify network states in the awake (and sleeping) brain that dates back to the earliest neural recordings in animals and humans. For example, Hans Berger used “extracellular” EEG recordings to characterize state-dependent alpha vs. beta waves in the awake brain of humans. Another more recent example was the identification of an increase in gamma activity in the primary visual cortex of awake mice during locomotion by Niell and Stryker, which they identified using LFP recordings. EEG and LFP recordings have been more often used than intracellular recordings to identify and classify network states in animals. The authors may consider using a slightly different language here (e.g. the membrane potential of neurons, which can be measured via intracellular recording techniques, has been shown to serve as a particularly sensitive indicator for network activity and has been instrumental in defining and characterizing network states in unprecedented detail). As the author demonstrate in Fig 4a, there is a high heterogeneity of the membrane activity from one recording to another. However, it is not clear if this variability is due to the recorded neurons (intrinsic properties [see remark line 212-213 which seems to indicate that the membrane resistance might differ between neurons] or location in the network [layers, functional connectivity, distance to the LFP electrode]) or due to the variability of the behavioral or internal context (arousal, locomotion, whisking...)? It would be important to settle this issue at the beginning of the paper. Indeed, if for similar LFP characteristics, neurons present a high heterogeneity of activities, it would be illusory to try to define an LFP index that would indicate the state of membrane fluctuation of the local neurons.

2) The behavioral context of the recordings is not characterized. It would make sense to determine the NSI associated with locomotion, pupil size, whisking as those behavioral contexts have been associated with specific membrane potential dynamics in cortical neurons. Were the headfixed mice allowed to run on top of a wheel, or was their movement restricted? Was there any task associated with these recordings? These may provide a richer dataset in which to define network state.

3) The goal behind this study is to emancipate research teams from having to perform intracellular recordings. Indeed, those experiments are complex and extremely low yield in most hands. To achieve this goal, it would be important to show that the proposed method generalizes to recordings of other labs performed in slightly different conditions. Indeed, all the parameters of the NSI have been optimized for the authors’ database (14 neurons). Thus, it is possible that the model is overfitting the data and cannot generalize to the LFP recordings of other labs. As the authors intend to share their software publicly, it would be good to first validate the approach using a more diverse dataset including different methods of LFP recording and in different animal models.

4) The authors state that the mean duration of the whole-cell recordings were 5.1 {plus minus} 2 min. Recordings of 3 min seem quite short in order to get a variety of states and the membrane potential is often “artificially” hyperpolarized in the beginning of recordings (first few minutes, depending on how much positive pressure was used to lower the recording electrode into the brain). Were these hyperpolarized periods included in the analysis? If so, this information should be included in the manuscript.

5) What was the distance between the patch clamp electrode and the LFP electrode during the recordings? The correlation between LFP and membrane potential recordings depends on distance (see e.g. Arroyo, Bennett and Hestrin, 2018). Could differences in the distance between the recording electrodes account for some of the differences in correlation shown in Fig.4? This issue should at least be briefly discussed in the manuscript.

6) The authors demonstrate that the gamma to delta ratio is not good to differentiate between non-rhythmic hyperpolarized states and rhythmic states. (line 251- 253) but they also seem to show in Figure 4 that the NSI is not accurate either (see Fig 4 and table 2). Intuitively, it would seems that the NSI and the gamma to delta ratio are both relying on the relationship between gamma and delta frequency as the NSI rely on the low frequency content (2-4 Hz) of the pLFP which is the temporally smoothed high gamma envelop of the LFP. The large amount of misclassification for the non-rhythmic hyperpolarized states is a serious problem for the NSI as the rhythmic state (characterized by 2-4 Hz oscillations) and the tonically depolarized state (characteristic of the locomotion state) can be easily detected in the LFP without complex analytical methods. Therefore, the strength of the model should be the classification of the bottom of the “U-model” of network states described by McGinley and colleagues (2015).

Minor Issues:

1) Line 535: “Overall, the dataset was dominated by non-rhythmic activity with a fraction of non-rhythmic states”.

2) We were not able to find the formula # 1 in the cited literature (Mallat, 1999).

3) “Low pupil diameter” should be “small pupil diameter” and “high pupil diameter” should be “large pupil diameter”.

4) If “Mukovski et al 2006” was indeed published online in March 2006, the paper actually belongs to an issue of Cerebral Cortex published in 2007 so I guess it should be “Mukovski et al 2007”.

5) It seems that the temporal width of the sliding window used to compute Y(t) was not optimized. Why?

6) Line 406 and 408. ratio -> ration.

7) Line 436 and Fig 4a. Some of the red circles indicate mismatch but other don’t (for i and iii). Maybe a different color for match and mismatch would facilitate the comprehension of the reader.

8) Line 492: why only 13 recordings (instead of 14) were used for the analysis reported here?

9) 546-548: Using a measure of the effect size in this case will be more informative.

10) Figure 1a: Reducing the thinness of the line so we can appreciate better the good quality of your recordings.

11) Figure 1d,e,i: The colored squares indicating the gamma and delta bands are too light and are barely visible

12) Figure 1e,f,j: What exactly are indicated by the scale bars?

13) Lines 58-60. The authors may additionally include references by Otto Creutzfeld and by Mircea Steriade, which were the first to show a direct relationship between LFP and membrane potential dynamics.
