# Supplementary figures and images for: Network States Classification based on Local Field Potential Recordings in the Awake Mouse Neocortex
Source: eNeuro. 2022 Aug 19;9(4):ENEURO.0073-22.2022. doi: 10.1523/ENEURO.0073-22.2022 (PMC9395246; doi:10.1523/ENEURO.0073-22.2022)

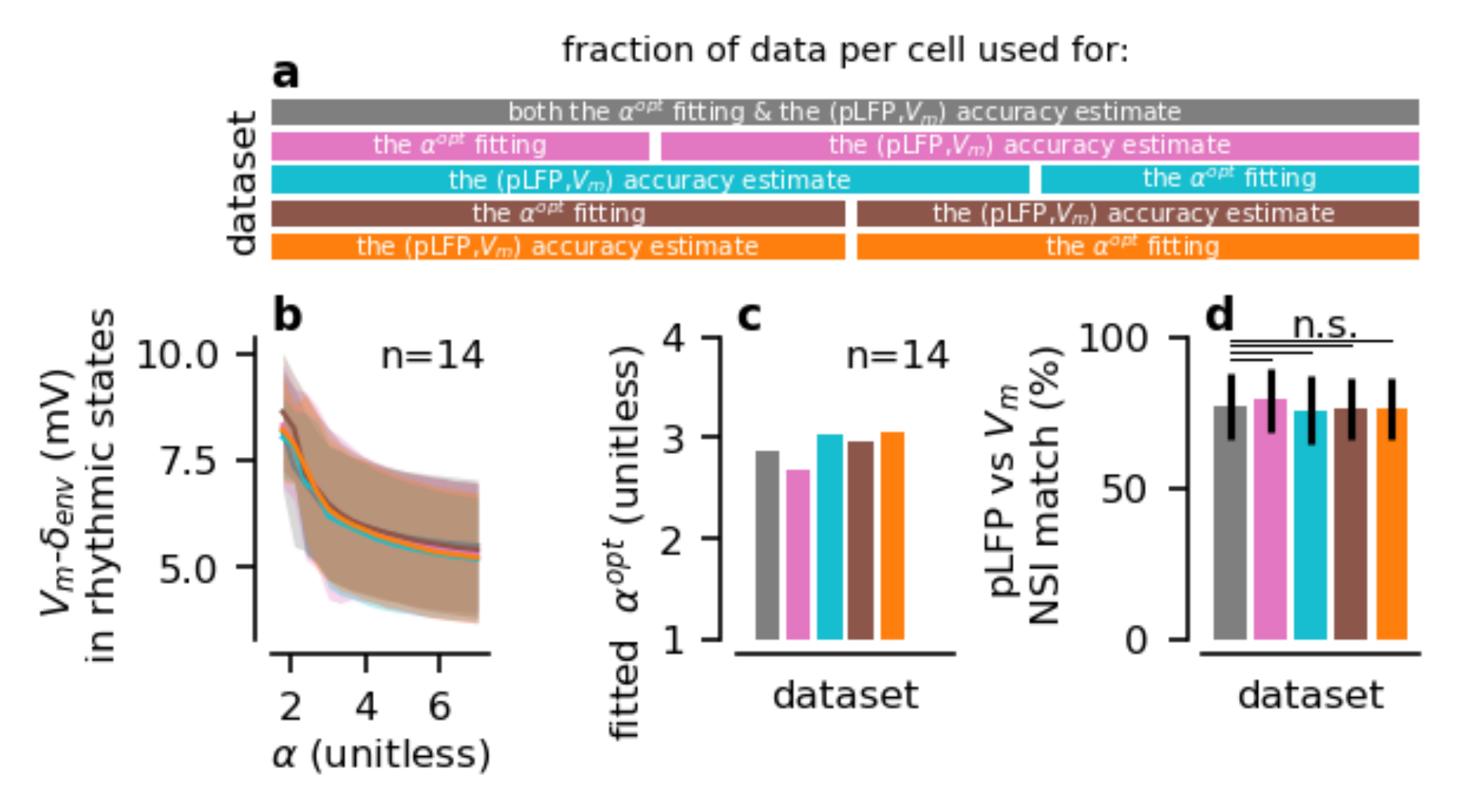

Supplement: Extended Data Figure 4-1 — Segmenting the dataset to perform the fitting and the accuracy estimate. a, We consider different segmentation for the analysis of the dataset: we fit and we estimate the accuracy on the full dataset (grey label, as in the main text), or we consider a given fraction of the data to fit and the rest of the dataset to estimate the accuracy of the classification (see the schematic of the dataset segmentation for the different cases considered). b, Mean envelope in the rhythmic states (see main text and Fig. 3c) as a function of the parameter for the different cases of dataset segmentation. We show the mean SEM over the n = 14 cells. c, Estimated obtained from the fitting of an exponential decay of the curves shown in b (see Materials and Methods and Fig. 3c). Note the narrow range of variations of (2.7–3.1) for the different cases of variations. d, Estimate of the NSI classification accuracy obtained from comparing the -defined and pLFP-defined NSI measure (see main text and Fig. 4c). We show the mean SEM over the n = 14 cells. All cases were found not to differ significantly from the accuracy reported in the main text, from left to right the different p = 0.12, p = 0.22, p = 0.52, p = 0.44 (paired student t test). Color code as in a. Download Figure 4-1, TIF file. [file enu-eN-MNT-0073-22-s02.tif]
